# MRI Radiomics Signatures of 21‐Gene Recurrence Score for Predicting Survival in ER+/HER2− Breast Cancer

**DOI:** 10.1002/cam4.71172

**Published:** 2025-09-02

**Authors:** Yang Chen, Lizhi Xie, Wei Tang, Qin Xiao, Li Liu, Tianwen Xie, Yan Huang, Qifeng Wang, Keda Yu, Yajia Gu, Weijun Peng

**Affiliations:** ^1^ Department of Radiology Fudan University Shanghai Cancer Center Shanghai China; ^2^ Department of Oncology, Shanghai Medical College Fudan University Shanghai China; ^3^ MR Research China GE Healthcare Beijing China; ^4^ Department of Pathology Fudan University Shanghai Cancer Center Shanghai China; ^5^ Department of Breast Surgery Fudan University Shanghai Cancer Center Shanghai China

**Keywords:** artificial intelligence & machine learning, breast cancer, magnetic resonance imaging (MRI), survival factors

## Abstract

**Background:**

Oncotype DX 21‐gene assays are recommended for evaluating distant recurrence and guiding decisions on the use of adjuvant therapy in ER+/HER2− breast cancers. However, it cannot be widely applied due to the high cost and time consumption.

**Purpose:**

To identify MRI radiomics signatures within tumor and peritumoral tissues associated with the 21‐gene recurrence score (RS) and explore their value in predicting 5‐year recurrence in young women with ER+/HER2− breast cancer.

**Methods:**

Two datasets were analyzed; all the enrolled patients were diagnosed with ER+/HER2− breast cancers and underwent preoperative breast MRIs. The radiogenomic development dataset, with RS data obtained from April 2017 to March 2019, was used to identify optimal RS‐signatures based on tumoral, peritumoral, and dilation radiomics features as well as clinical‐imaging characteristics with the support vector machine method. The prognosis dataset, in which patients aged 18 to 40 years received breast surgery followed by adjuvant chemotherapy from 2012 to 2016, was used to evaluate the prognostic implication of the proposed optimal RS‐signatures by measuring 5‐year disease‐free survival (DFS) with the Cox proportional hazards model.

**Results:**

159 patients (111 in the training and 48 in the validation groups) and 111 young patients (a mean follow‐up time of 49.6 months and a 5‐year DFS of 83.8%) were enrolled in these datasets, respectively. Areas under the receiver operating characteristic curve (AUCs) of the three optimal RS‐signatures were 0.74 (95% CI: 0.59–0.87), 0.75 (95% CI: 0.61–0.88) and 0.74 (95% CI: 0.59–0.87) respectively. In the prognosis dataset, there were significant differences in survival between the patients in the predicted high‐risk and low‐risk groups categorized by the above three signatures, and the predicted recurrence risks were independent factors for DFS.

**Conclusion:**

The radiomic signatures within the tumor and peritumoral region exhibited potential to guide decisions on the use of chemotherapy and predict survival.

## 1 Introduction

Oncotype DX 21‐gene assays are recommended by the National Comprehensive Cancer Network (NCCN) guidelines for evaluating distant recurrence and guiding decisions on the use of adjuvant systemic therapy in ER+/HER2‐ patients [1]. The 3.2024 version of NCCN guidelines (available at NCCN.org) recommends that patients be stratified into low, medium, and high recurrence risk groups using the 21‐gene assay recurrence score (RS) of 15 and 26 as the cutoffs [1]. High‐risk patients benefit from adjuvant chemotherapy; low‐risk patients are recommended to be exempted from chemotherapy, and intermediate‐risk patients need to make decisions based on comprehensive clinical information. The 21‐gene test is currently not widely used in the clinic because it is costly and time‐consuming.

Breast MRI is the most sensitive technology for diagnosis and treatment response evaluation in breast cancers [2, 3, 4, 5]. Extracting richer information from MRI images, such as objective quantitative features, instead of traditional subjective MR imaging features, is now possible due to the development of radiomics. What is more, the tumor microenvironment has been shown to play an important role in the development and progression of breast cancer [6, 7], so the region of interest in radiomics extends from the tumor to the peritumoral tissues. A few studies, which tried to identify MRI‐based biomarkers of RS, reported potential correlations between the RS and traditional MR imaging and tumoral and peritumoral radiomic features [8, 9, 10, 11, 12, 13, 14]. However, among the studies mentioned above, those adopting the latest RS cutoff are very limited [10, 11]. Additionally, some studies included patients with ER− or HER2+ breast cancer who do not meet the current guideline‐recommended application population [8, 9, 13, 14]. Studies involving T2WI sequences are rare and very few studies focused on the peritumoral environment [10, 11].

On the other hand, although few studies have established RS‐prediction models based on radiomic characteristics [11, 14], the associations of these models with realistic clinical survival remain unclear. Only one study by Fan et al. [15] demonstrated that the RS‐prediction model has potential value in evaluating the efficacy of neoadjuvant therapy. To our knowledge, there has never been a published study exploring its value in assessing survival after adjuvant chemotherapy in young patients.

Therefore, the objectives of this study were (1) to identify MRI radiomics signatures associated with RS within tumor and peritumoral tissues in patients with ER+/HER2− breast cancer and (2) to estimate the value of the proposed signatures in assessing survival after adjuvant chemotherapy in young women.

## 2 Materials and Methods

### 2.1 Dataset

**FIGURE 1:**
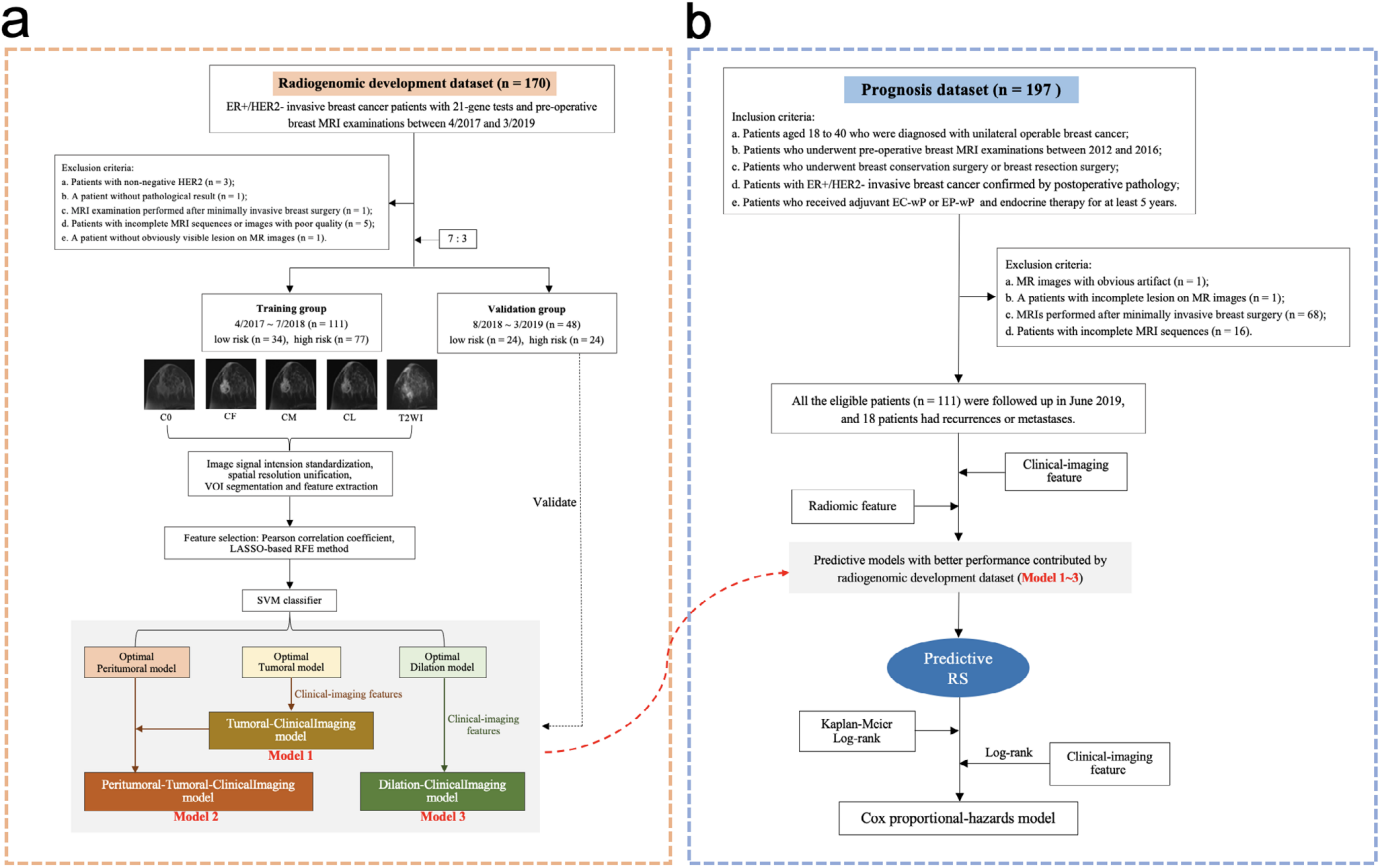
Patient selection in the radiogenomic development dataset (a) and prognosis dataset (b), with study frameworks of the RS predictive model construction and prognostic assessment of these models. The pre‐ (C0), first‐ (CF), middle (CM) and last‐enhanced (CL) phases of dynamic contrast‐enhanced (DCE) images.

This retrospective study was approved by the ethics committee of Fudan University Shanghai Cancer Center (2203252‐23), and informed consent was waived. Two independent datasets were involved: the radiogenomic development dataset and the prognosis dataset. The radiogenomic development dataset was used to identify radiomics signatures associated with the recurrence risk quantified by RS, obtaining a model to predict recurrence risk, while clinical, MR imaging, and radiomic characteristics of prognosis dataset patients were applied to verify the prognostic value of the proposed models in young women who did not perform 21‐gene tests. The study framework is shown in Figure 1.

#### 2.1.1 Radiogenomic Development Dataset

Patients with invasive breast cancer who underwent preoperative MRI examination and Oncotype DX 21‐gene assay in our hospital from April 2017 to March 2019 consecutively were screened (*n* = 170) for the radiogenomic development dataset, and clinicopathological information and MRI data were collected. The patients were divided into a training group and a validation group according to the time order and ratio of 7:3 (Figure 1a).

#### 2.1.2 Prognosis Dataset

The prognosis dataset included patient data from the SPECTRUM (Substitution of paclitaxel for cyclophosphamide on survival outcomes and resumption of menses in young women with ER‐positive breast cancer) trial [16]. Patients aged 18 to 40 years and diagnosed with unilateral operable breast cancer who underwent breast conservation surgery or mastectomy at our hospital from 2012 to 2016 were screened. All the patients were ER+/HER2− confirmed by postoperative pathology. Patients with preoperative breast MRI data who received adjuvant epirubicin/cyclophosphamide followed by weekly paclitaxel (EC‐wP) or epirubicin/paclitaxel followed by weekly paclitaxel (EP‐wP), followed by endocrine therapy for at least 5 years were enrolled (*n* = 197) (Figure 1b).

#### 2.1.3 Exclusion Criteria for the Two Datasets

The exclusion criteria for the two datasets were as follows: had positive or equivocal HER2 expression; no pathological information; poor image quality; and nonnegligible movement artifacts; MRI sequences were incomplete; lesions were not clearly visible on the MR images; lesions on the MR images were incomplete, and parts of the lesions were out of range on the MR images; and MRIs were obtained after minimally invasive breast surgery.

#### 2.1.4 Follow‐Up of the Prognosis Dataset

All the patients in the prognosis dataset were followed up in June 2019. The follow‐up information was as follows: (a) whether recurrence or metastasis occurred, and if so, when recurrence or metastasis occurred; (b) whether there was a second primary breast cancer or other second primary malignant tumor, and if so, the first time of detection; and (c) whether the patient died, and if so, the cause and time of death. Disease‐free survival (DFS) was defined as the time from the beginning of postoperative chemotherapy to the occurrence of recurrence, metastasis, second primary breast cancer, other second primary cancers or death.

### 2.2 Clinical Characteristics

Clinical information of the patients in the radiogenomic development dataset was obtained from the electronic medical record system, including age, body mass index, and whether they were postmenopausal. We also obtained their pathology type, progesterone receptor (PR) status, tumor size, invasive malignancy grade, whether they had concomitant ductal carcinoma in situ, and whether they had lymph node metastasis from the pathology report.

For the prognosis dataset, age, type of surgery, chemotherapy regimen, whether or not they received radiotherapy, PR status, tumor size, invasive malignancy grade, and whether or not they had lymph node metastasis were used in the analysis.

### 2.3 MRI Protocol

The MRI scans were completed under three different MRI scanners, and the scanning parameters are shown in Table S1. During examination, patients were in a prone position, and both breasts were naturally placed in a 16‐channel mammary coil. The MRI examinations included T2WI and dynamic contrast enhancement (DCE) in the transverse plane, T2WI used turbo spin–echo or short time inversion recovery sequence, and DCE was obtained using T1‐weighted volume imaging for breast assessment (VIBRANT) or 3D gradient‐echo volumetric interpolated breath‐hold examination (VIBE) sequence. After a plain scan, the contrast agent was injected (Magnevist, Bayer HealthCare Pharmaceuticals Inc., Wayne, USA) at a rate of 1.5–2.0 mL/s at a dose of 0.1 mmol/kg, followed by a 20 mL saline flush. The first phase of enhanced images was collected at 30–46 s after the injection of the contrast agent; then, 3–4 phases of enhanced scanning images were obtained continuously without intervals, and the image acquisition time of each phase was 38–40 s.

### 2.4 Recurrence Score

All enrolled patients of the radiogenomic development dataset underwent a 21‐gene expression assay, and the RS was obtained at our hospital. The RS ranged from 0 to 100; the greater the RS, the higher the 10‐year recurrence risk and the greater the benefit of chemotherapy. According to the 3.2024 version of the National Comprehensive Cancer Network (NCCN) clinical practice guidelines for breast cancer (available at NCCN.org) [1], this study used 26 as the cutoff value and divided the patients into a low‐risk group (RS < 26) and a high‐risk group (RS ≥ 26).

### 2.5 MR Imaging Feature Evaluation

Two experienced breast radiologists (with 11 and 6 years of experience in breast imaging diagnosis) reviewed and evaluated the patients' MR images according to the 2013 version of the Breast Imaging Reporting and Data System (BI‐RADS) lexicon [17]. The amount of fibroglandular tissue was divided into extreme fibroglandular tissue, heterogeneous fibroglandular tissue, scattered fibroglandular tissue, and almost entirely fat. The degree of background parenchymal enhancement was divided into four categories: minimal, mild, moderate, and marked. MRI findings included non‐mass enhancement (NME) and mass, and their enhancement curve type (persistent, plateau type, or washout) and BI‐RADS classification were evaluated. If MRI showed a mass, it was necessary to describe the shape (round, oval, or irregular), margin (circumscribed, irregular, or spiculated), and internal enhancement characteristics (homogeneous, heterogeneous, rim enhancement, or dark internal septation). If an NME was observed, its internal enhancement characteristics (homogeneous, heterogeneous, clumped or clustered ring) and distribution (focal, linear, segmental, regional, multiple regions or diffuse) were recorded.

If the evaluation results of these two radiologists were inconsistent, a third senior radiologist (with 21 years of experience in breast imaging diagnosis) reviewed the images and determined the final results. The three radiologists knew that all the lesions were invasive breast cancers, but they were blind to the RSs.

### 2.6 Volume of Interest Segmentation

A radiologist (6 years of experience) manually delineated the lesion contour layer by layer in the last phase of post‐enhancement (CL) images to obtain the tumoral volume of interest (VOI), with all lesions included. Then, the VOI was copied onto T2W, pre‐enhancement (C0), first phase of post‐enhancement (CF), and middle phase of post‐enhancement (CM) images. The VOIs were checked one by one, and manual adjustments were made for those that did not match the lesion well. Open source software (ITK‐SNAP, version 3.8.0, http://www.itksnap.org/) was used for manual VOI segmentation and adjustment, and VOI replication was completed in Python (version 3.8).

**FIGURE 2:**
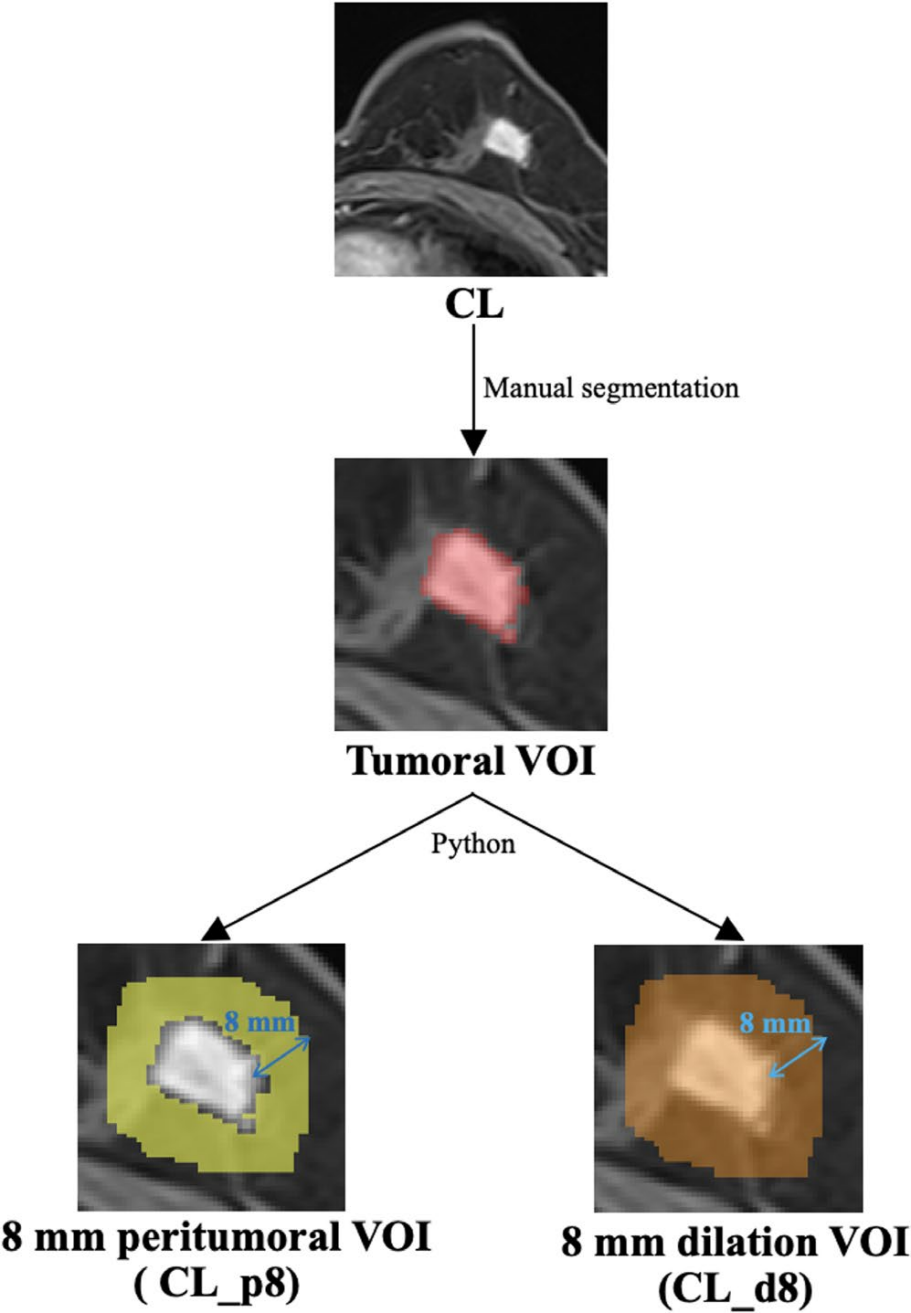
Volume of interest (VOI) segmentation. First, a tumoral VOI was delineated manually on the last‐enhanced (CL) phase of dynamic contrast‐enhanced images. Then the 8 mm peritumoral and dilation VOIs were automatically obtained using Python.

On the basis of the tumoral VOI, Python 3.8 was used to automatically dilate the VOI contour outward, with dilation ranges of 2, 4, 6, 8, and 10 mm. In this way, dilated VOIs (tumor + circular peritumor regions of 2, 4, 6, 8, and 10 mm) were obtained. By subtracting the tumoral VOI from the dilated VOI, the peritumoral VOI could be obtained (circular areas of 2, 4, 6, 8, and 10 mm around the tumor, excluding the internal region of the tumor). Check each dilated and peritumoral VOI, and manually adjust the VOI beyond the breast contour. An example of the VOI segmentation is shown in Figure 2.

### 2.7 Image Preprocessing and Radiomic Feature Extraction

Because the MRI examinations were obtained with three different MRI scanners, the scanning parameters were not completely consistent. Before feature extraction, the image signal intensity was standardized, and the spatial resolution was unified. (1) The formula for image signal intensity standardization was as follows:

$$I_{\mathrm{normalize}}=\frac{I-\mu }{\sigma }\times 100$$

I_normalize_ is the normalized image gray value, I is the original image signal intensity, and μ and σ are the mean value and standard deviation of I, respectively. (2) Using the cubic spline interpolation algorithm, the spatial resolution of the DCE images (including C0, CF, CM and CL) was resized to 0.9 × 0.9 × 2.2 mm, and the spatial resolution of the T2W images was resized to 1.2 × 1.2 × 7 mm.

After image preprocessing, 1046 features were extracted from each VOI, including 100 original image features, 258 Laplacian of Gaussian (LoG) image features, and 688 wavelet image features. LoG features were extracted by filtering the original images with a LoG filter with δ values of 2.0, 3.0, and 4.0. We also filtered the original images with a wavelet filter to extract the wavelet features. The bin width was set to 5. The Python package from PyRadiomics (v1.3.1, PyRadiomics Community) was used to extract features, and all other parameters were set to their default values.

### 2.8 Feature Selection and Radiomic Model Construction

All features were standardized by the *z* score method, and the validation group features were standardized using the average and standard deviation of the training group features. To reduce multicollinearity between features, Pearson correlation coefficient screening and recursive feature elimination (RFE) methods were implemented. First, the Pearson correlation coefficient was calculated between every feature pair. For those with a Pearson correlation coefficient > 0.9, the mean Pearson correlation coefficient was calculated between each of them and all other features, and the feature with the higher mean Pearson correlation coefficient was removed. The base estimator for RFE feature filtering was the least absolute shrinkage and selection operator (LASSO) algorithm, and α was set to the default value of 1. The upper limit for feature number was 10% of the sample size, and the feature set with the highest model accuracy was selected as the optimal one.

Due to the imbalanced distribution between the low‐risk and high‐risk groups in the training group, the synthetic minority oversampling technique (SMOTE) was used to resample the training group before establishing the model, while the data from the validation group patients were not resampled. Then, an RS‐prediction model was constructed using the linear support vector machine (SVM) method. The predictive performance of the model was evaluated by receiver operating characteristic (ROC) curve analysis. The data processing above was completed in Python (version 3.8).

#### 2.8.1 Tumoral Model Construction

DCE (C0, CF, CM, CL) and T2 sequences were used to construct tumoral models. The optimal tumoral model was fused with clinical‐imaging features to obtain a fusion model (Model 1).

#### 2.8.2 Construction of the Peritumoral Model

Peritumoral models were constructed based on the features of CL and T2W images, and the optimal peritumoral model was selected to be fused with “Model 1” to construct a fusion model (Model 2), which included peritumoral, tumoral, and clinical‐imaging information. In the model fusion process, a logistic regression analysis was performed by combining the predicted probability of the optimal peritumoral model with that of Model 1.

#### 2.8.3 Dilation Model Construction

The dilation classifiers were developed using features from dilated VOIs on CL, T2W images and their combination. The optimal dilation model was subsequently selected and combined with clinical‐imaging features to generate a fusion model (Model 3).

### 2.9 Assessment of the Prognostic Implications of Radiogenomic Signatures

We identified predictive RSs (predRSs) of the prognosis dataset by incorporating their characteristics into the top 3 predictive models (Model 1–3).

The Kaplan–Meier method was used to analyze the survival of patients in different predRS groups; the log‐rank test was performed to determine whether differences existed between the predRS groups. The univariate log‐rank test was used to select supplementary clinical‐imaging features related to the DFS, which were incorporated into the Cox proportional hazards model as well as the predRS, with duplicate features removed. The Cox model was visualized and evaluated by a forest plot, nomogram, and calibration curve.

A *p* value < 0.05 was considered to indicate statistical significance. The relevant data processing and statistical plotting were performed using Python (version 3.8), GraphPad Prism (version 9.5.0), and SPSS software (version 25.0).

## 3 Results

### 3.1 Clinical and MR Imaging Characteristics of the Radiogenomic Development Dataset

According to the chronological order and 7:3 ratio, patients in the radiogenomic development dataset who underwent the 21‐gene assay from April 2017 to July 2018 were classified into the training group, including a total of 111 patients, 34 at low risk and 77 at high risk. Patients who underwent the 21‐gene assay testing from August 2018 to March 2019 were included in the validation group, which included 48 patients, 24 at low risk and 24 at high risk. In the training group, the average age of the low‐risk patients was significantly higher than that of the high‐risk patients, and there was a significant difference in the tumor margins between patients with different recurrence risks (Tables 1 and 2). The other clinical features and imaging characteristics were not significantly different (Tables 1 and 2).

**TABLE 1:** Characteristics of patients in radiogenomic development dataset.

Characteristics	Training group (*n* = 111)			Validation group (*n* = 48)		
	Low recurrence risk (*n* = 34)	High recurrence risk (*n* = 77)	*p*	Low recurrence risk (*n* = 24)	High recurrence risk (*n* = 24)	*p*
Age	55.0 ± 9.5^a^	51.1 ± 8.9^a^	0.041^b^	51.2 ± 8.2^a^	52.7 ± 8.6^a^	0.528
Body mass index	23.1 ± 2.9^a^	23.3 ± 2.5^a^	0.640	23.1 ± 2.6^a^	23.5 ± 2.9^a^	0.670
Postmenopausal	20 (58.8%)	40 (51.9%)	0.503	13 (54.2%)	14 (58.3%)	0.771
Tumor size	34	77	0.930	24	24	1.000
pT1	25 (73.5%)	56 (72.7%)		15 (62.5%)	15 (62.5%)	
pT2/3	9 (26.5%)	21 (27.3%)		9 (37.5%)	9 (37.5%)	
Pathology	34	77	0.226	24	24	0.109
Invasive ductal carcinoma	28 (82.4%)	71 (92.2%)		24 (100.0%)	20 (83.3%)	
The other types of invasive carcinomas^c^	6 (17.6%)	6 (7.8%)		0 (0)	4 (16.7%)	
Invasive malignancy grade	34	77	0.955	24	24	0.609
Grade I/II	30 (88.2%)	66 (85.7%)		23 (95.8%)	21 (87.5%)	
Grade III	4 (11.8%)	11 (14.3%)		1 (4.2%)	3 (12.5%)	
DCIS present	5 (14.7%)	7 (9.1%)	0.585	5 (20.8%)	5 (20.8%)	1.000
Progesterone receptor positive	34 (100%)	70 (90.9%)	0.098	23 (95.8%)	18 (75.0%)	0.097
Negative lymph node	28 (82.4%)	66 (85.7%)	0.650	21 (87.5%)	16 (66.7%)	0.086

Abbreviation: DCIS, ductal carcinoma in situ.

^a^ Data are means ± standard.

^b^ Means statistically significant.

^c^ The other types of invasive carcinomas include invasive lobular carcinoma, invasive micropapillary carcinoma, pure mucinous breast cancer, and mixed carcinoma including two different types.

**TABLE 2:** MR imaging features of lesions in radiogenomic development dataset.

Characteristics	Training group (*n* = 111)			Validation group (*n* = 48)		
	Low recurrence risk (*n* = 34)	High recurrence risk (*n* = 77)	*p*	Low recurrence risk (*n* = 24)	High recurrence risk (*n* = 24)	*p*
Amount of fibroglandular tissue	34	77	0.166	24	24	0.549
Almost entirely fat	2 (5.9%)	0 (0)		1 (4.2%)	0 (0)	
Scattered fibroglandular tissue	5 (14.7%)	11 (14.3%)		2 (8.3%)	5 (20.8%)	
Heterogeneous fibroglandular tissue	23 (67.6%)	50 (64.9%)		18 (75.0%)	15 (62.5%)	
Extreme fibroglandular tissue	4 (11.8%)	16 (20.8%)		3 (12.5%)	4 (16.7%)	
Background parenchymal enhancement	34	77	0.949	24	24	0.718
Minimal	20 (58.8%)	48 (62.3%)		13 (54.2%)	12 (50.0%)	
Mild	11 (32.4%)	22 (28.6%)		7 (29.2%)	9 (37.5%)	
Moderate	3 (8.8%)	7 (9.1%)		4 (16.7%)	2 (8.3%)	
Marked	0 (0)	0 (0)		0 (0)	1 (4.2%)	
MRI finding	34	77	1.000	24	24	1.000
Mass	33 (97.1%)	74 (96.1%)		21 (87.5%)	21 (87.5%)	
NME	1 (2.9%)	3 (3.9%)		3 (12.5%)	3 (12.5%)	
Mass shape	33	74	0.400	21	21	0.292
Round	2 (6.1%)	3 (4.1%)		0 (0)	0 (0)	
Oval	6 (18.2%)	23 (31.1%)		7 (33.3%)	4 (19.0%)	
Irregular	25 (75.8%)	48 (64.9%)		14 (66.7%)	17 (81.0%)	
Mass margin	33	74	0.013*	21	21	0.123
Irregular	8 (24.2%)	39 (52.7%)		8 (38.1%)	13 (61.9%)	
Circumscribed	3 (9.1%)	3 (4.1%)		0 (0)	0 (0)	
Spiculated	22 (66.7%)	32 (43.2%)		13 (61.9%)	8 (38.1%)	
Mass internal enhancement	33	74	0.764	21	21	1.000
Heterogeneous	25 (75.8%)	58 (78.4%)		18 (85.7%)	19 (90.5%)	
Rim enhancement	8 (24.2%)	16 (21.6%)		3 (14.3%)	2 (9.5%)	
NME distribution	1	3	NA	3	3	1.000
Segmental	1 (100%)	3 (100%)		2 (66.7%)	1 (33.3%)	
Regional or multiple regions	0 (0)	0 (0)		1 (33.3%)	2 (66.7%)	
NME internal enhancement	1	3	1.000	3	3	1.000
Heterogeneous	0 (0)	1 (33.3%)		1 (33.3%)	0 (0)	
Clumped or clustered ring	1 (100%)	2 (66.7%)		2 (66.7%)	3 (100%)	
Initial phase of kinetic curve	34	76	0.839	24	24	0.125
Fast	22 (64.7%)	50 (65.8%)		11 (45.8%)	5 (20.8%)	
Medium	11 (32.4%)	25 (32.9%)		13 (54.2%)	18 (75.0%)	
Slow	1 (2.9%)	1 (1.3%)		0 (0)	1 (4.2%)	
Delayed phase of kinetic curve	34	76	0.635	24	24	0.303
Persistent	2 (5.9%)	2 (2.6%)		0 (0)	0 (0)	
Plateau type	8 (23.5%)	23 (30.3%)		4 (16.7%)	7 (29.2%)	
Washout	24 (70.6%)	51 (67.1%)		20 (83.8%)	17 (70.8%)	
Assessment category	34	77	0.926	24	24	0.720
4A	1 (2.9%)	3 (3.9%)		0 (0)	0 (0)	
4B	9 (26.5%)	19 (24.7%)		4 (16.7%)	6 (25.0%)	
4C	18 (52.9%)	37 (48.1%)		16 (66.7%)	13 (54.2%)	
5	6 (17.6%)	18 (23.4%)		4 (16.7%)	5 (20.8%)	

Abbreviation: NME, non‐mass enhancement.

* Means statistically significant.

### 3.2 Performance of Radiogenomic Signatures

#### 3.2.1 Performance of Tumoral Models

For tumoral models, the CL‐T2 model had the highest performance, with an AUC of 0.71 in the validation group. The CL‐T2‐ClinicalImaging model (Model 1) was constructed by incorporating clinical‐imaging features into the CL‐T2 model; the performance of Model 1 was further improved, with an AUC of 0.74.

The Model 1 included a total of eight features, involving three features from T2W images (T2_log‐sigma‐4‐0‐mm‐3D_Gray‐Level Size Zone Matrix_SmallAreaLowGrayLevelEmphasis, T2_wavelet‐LLH_Gray‐Level Co‐occurrence Matrix_Idn and T2_wavelet‐LLL_Gray‐Level Dependence Matrix _LargeDependenceLowGrayLevelEmphasis), one feature from CL (CL_wavelet‐HHL_firstorder_Kurtosis), and four clinical‐imaging features (PR status, mass margin, amount of fibroglandular tissue and NME internal enhancement).

**FIGURE 3:**
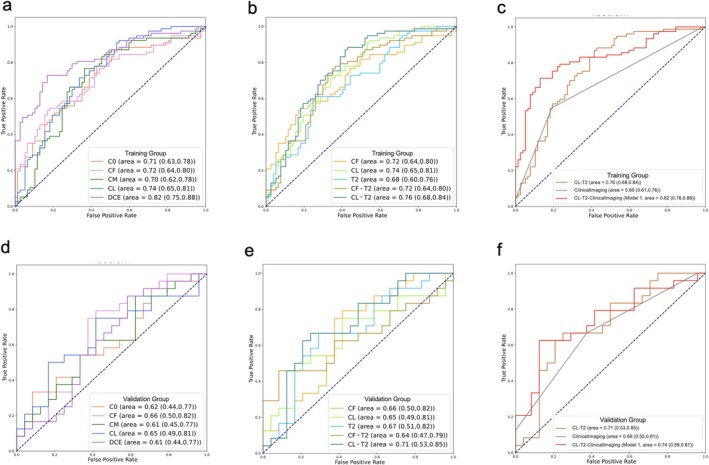
Receiver operating characteristic (ROC) curves of different tumoral models for differentiating between low (RS < 26) and high recurrence risk (RS ≥ 26) ER+/HER2+ breast cancer patients in the training (a–c) and validation (d–f) groups.

The performance of each tumoral model is shown in Figure 3 and Table S2, and the incorporated radiomic features are shown in Figure S1.

#### 3.2.2 Performance of Peritumoral Models

**FIGURE 4:**
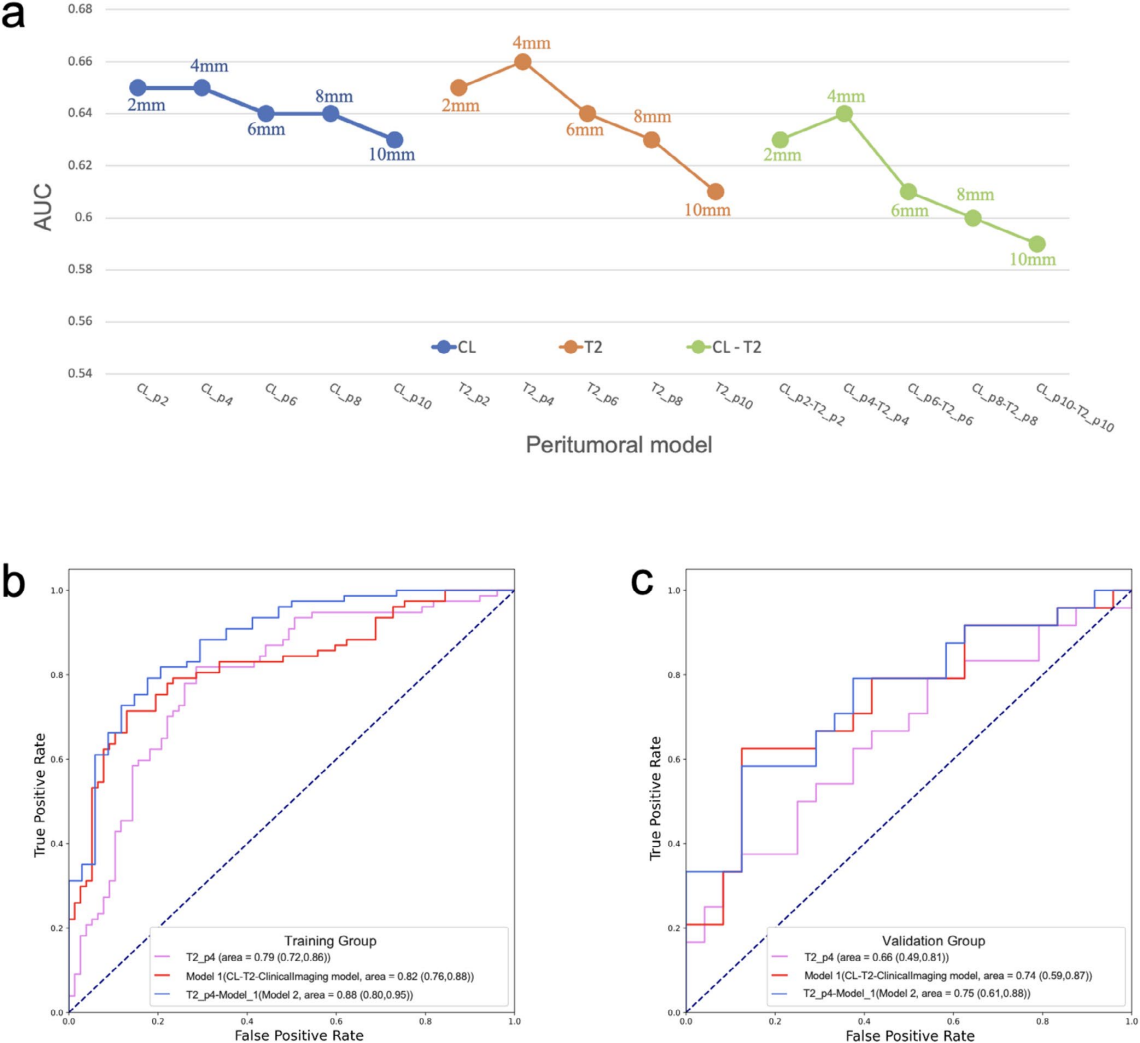
Change trend in peritumoral model performance in the validation group, and the optimal peritumoral model based on 4 mm peritumoral region on T2W images (T2_p4) (a). Receiver operating characteristic (ROC) curves of the T2_p4, the optimal tumoral‐clinical imaging model (CL‐T2‐ClinicalImaging), and their fusion model (Model 2) (b, c).

Among the 15 peritumoral models in the validation group, the highest AUC was achieved by the model within the 4 mm peritumoral region on T2W images (T2_p4), with an AUC of 0.66 (95% CI = 0.49–0.81) and an accuracy, sensitivity, and specificity of 0.60, 0.67, and 0.54, respectively (Figure 4a and Table S3).

The T2_p4 model was combined with Model 1 to construct a fusion model (Model 2). In the validation group, the AUC of Model 2 was 0.75, which was higher than that of the T2_p4 model (AUC = 0.66) and Model 1 (AUC = 0.74) (Figure 4b,c).

The features included in these models are shown in Appendix S1.

#### 3.2.3 Performance of the Dilation Models

Among the 15 dilation models, the AUC for the 4 mm dilation region on CL (CL_d4) was highest in the validation group (0.69, 95% CI = 0.51–0.82) (Table 3 and Figure S2a).

**TABLE 3:** Performance of dilation models.

Dilation model	Training group				Validation group			
	AUC (95% CI)	Accuracy	Sensitivity	Specificity	AUC (95% CI)	Accuracy	Sensitivity	Specificity
CL_d2	0.82 (0.74–0.88)	0.75 (0.69–0.82)	0.65 (0.59–0.79)	0.86 (0.75–0.91)	0.67 (0.52–0.82)	0.63 (0.48–0.75)	0.75 (0.57–0.91)	0.50 (0.26–0.67)
CL_d4	0.76 (0.67–0.83)	0.70 (0.64–0.77)	0.70 (0.60–0.80)	0.71 (0.61–0.81)	**0.69 (0.51–0.82)**	0.58 (0.44–0.71)	0.79 (0.61–0.95)	0.38 (0.19–0.56)
CL_d6	0.93 (0.88–0.96)	0.82 (0.73–0.86)	0.83 (0.70–0.88)	0.81 (0.71–0.89)	0.65 (0.49–0.81)	0.63 (0.50–0.79)	0.71 (0.53–0.88)	0.54 (0.38–0.79)
CL_d8	0.88 (0.82–0.93)	0.82 (0.75–0.88)	0.78 (0.68–0.87)	0.86 (0.77–0.93)	0.64 (0.48–0.79)	0.44 (0.29–0.58)	0.42 (0.22–0.63)	0.46 (0.27–0.67)
CL_d10	0.82 (0.76–0.89)	0.73 (0.66–0.80)	0.65 (0.54–0.76)	0.81 (0.71–0.89)	0.64 (0.48–0.80)	0.54 (0.42–0.69)	0.67 (0.47–0.86)	0.42 (0.25–0.65)
T2_d2	0.84 (0.78–0.90)	0.76 (0.69–0.83)	0.74 (0.65–0.85)	0.78 (0.67–0.86)	0.67 (0.52–0.83)	0.58 (0.44–0.73)	0.75 (0.55–0.93)	0.42 (0.23–0.63)
T2_d4	0.83 (0.77–0.89)	0.79 (0.71–0.84)	0.75 (0.66–0.85)	0.83 (0.71–0.89)	0.68 (0.53–0.83)	0.63 (0.48–0.75)	0.88 (0.74–1.00)	0.38 (0.17–0.57)
T2_d6	0.71 (0.63–0.79)	0.69 (0.61–0.76)	0.64 (0.53–0.74)	0.74 (0.63–0.83)	0.65 (0.48–0.80)	0.63 (0.48–0.73)	0.79 (0.57–0.91)	0.46 (0.27–0.67)
T2_d8	0.66 (0.57–0.74)	0.56 (0.48–0.64)	0.48 (0.35–0.58)	0.64 (0.54–0.75)	0.65 (0.49–0.79)	0.63 (0.48–0.75)	0.46 (0.24–0.67)	0.79 (0.63–0.94)
T2_d10	0.70 (0.62–0.78)	0.62 (0.55–0.70)	0.65 (0.53–0.75)	0.60 (0.51–0.72)	0.62 (0.46–0.78)	0.63 (0.50–0.77)	0.71 (0.52–0.88)	0.54 (0.35–0.74)
CL_d2‐T2_d2	0.82 (0.75–0.88)	0.71 (0.65–0.79)	0.65 (0.57–0.78)	0.77 (0.67–0.86)	0.63 (0.47–0.78)	0.63 (0.44–0.73)	0.63 (0.42–0.83)	0.63 (0.36–0.75)
CL_d4‐T2_d4	0.85 (0.78–0.91)	0.81 (0.71–0.84)	0.82 (0.67–0.86)	0.79 (0.70–0.88)	0.65 (0.49–0.80)	0.56 (0.42–0.71)	0.88 (0.73–1.00)	0.25 (0.08–0.42)
CL_d6 ‐T2_d6	0.89 (0.84–0.94)	0.85 (0.79–0.90)	0.77 (0.68–0.87)	0.94 (0.85–0.97)	0.62 (0.45–0.78)	0.60 (0.48–0.75)	0.62 (0.48–0.85)	0.58 (0.33–0.75)
CL_d8‐T2_d8	0.86 (0.80–0.92)	0.81 (0.74–0.86)	0.77 (0.67–0.86)	0.84 (0.76–0.92)	0.62 (0.46–0.80)	0.63 (0.48–0.75)	0.54 (0.35–0.75)	0.71 (0.50–0.88)
CL_d10‐T2_d10	0.78 (0.70–0.86)	0.71 (0.64–0.79)	0.65 (0.52–0.72)	0.78 (0.72–0.90)	0.60 (0.43–0.77)	0.63 (0.48–0.75)	0.67 (0.47–0.85)	0.58 (0.39–0.77)

*Note:* CL_d2 ~ CL_d10: dilation models based on features extracted from 2 mm (d2), 4 mm (d4), 6 mm (d6), 8 mm (d8), 10 mm (d10) dilation ranges on the last‐enhanced (CL) phases of dynamic contrast‐enhancement (DCE) images. T2_d2 ~ T2_d10: dilation models based on features extracted from 2 mm (d2), 4 mm (d4), 6 mm (d6), 8 mm (d8), 10 mm (d10) dilation ranges on the T2W images. CL_d2‐T2_d2 ~ CL_d10‐T2_d10: dilation models based on features extracted from 2 mm (d2), 4 mm (d4), 6 mm (d6), 8 mm (d8), 10 mm (d10) dilation ranges on the CL and T2W images. The bolded values indicates the maximum AUC value for the validation group in Table 3.

Clinical‐imaging features were incorporated into CL_d4 model. The AUC of CL_d4‐ClinicalImaging (Model 3) was 0.74, which was higher than those of CL_d4 (AUC = 0.69) and ClinicalImaging (AUC = 0.68) models (Figure S2b,c).

The features included in the dilation models are shown in Appendix S2.

### 3.3 Characteristics of the Prognosis Dataset

A total of 111 patients were included in the prognosis dataset, whose clinical‐imaging characteristics are summarized in Table 4. The mean follow‐up time was 49.6 months (range 10–84 months); a total of 18 patients (16.2%) experienced recurrence, metastasis, or death, with a mean disease‐free survival (DFS) of 30.9 months (range 10–60 months). Three of them experienced local recurrence, 11 had distant metastasis, two had contralateral primary breast cancer, one had a second primary malignancy, and one died.

**TABLE 4:** Univariate log‐rank test.

Characteristic	Number (%)	χ^2^	*p*
Age		1.149	0.284
≤ 35	48 (43.2%)		
> 35	63 (56.8%)		
Tumor size		5.737	0.017^a^
pT1	53 (47.7%)		
pT2/3	58 (52.3%)		
Invasive malignancy grade		1.684	0.194
I/II	68 (61.3%)		
III	43 (38.7%)		
Progesterone receptor status		0.957	0.328
Positive	93 (83.8%)		
Negative	18 (16.2%)		
Lymph node		0.673	0.412
Positive	74 (66.7%)		
Negative	37 (33.3%)		
Type of surgery		6.329	0.043^a^
Breast conservative surgery	32 (28.9%)		
Mastectomy	79 (71.2%)		
Radiotherapy		0.012	0.912
Yes	70 (63.1%)		
No	41 (36.9%)		
Chemotherapy regimen		0.003	0.959
EC‐wP	44 (39.6%)		
EP‐wP	67 (60.4%)		
Amount of fibroglandular tissue		3.951	0.267
Almost entirely fat	0 (0)		
Scattered fibroglandular tissue	6 (5.4%)		
Heterogeneous fibroglandular tissue	74 (66.7%)		
Extreme fibroglandular tissue	31 (27.9%)		
Degree of background parenchymal enhancement		6.294	0.098
Minimal	47 (42.3%)		
Mild	36 (32.4%)		
Moderate	25 (22.5%)		
Marked	3 (2.7%)		
Mass shape		4.907	0.179
Round	1 (1.3%)		
Oval	37 (46.8%)		
Irregular	41 (51.9%)		
Mass margin		6.24	0.044^a^
Irregular	38 (48.1%)		
Circumscribed	3 (3.8%)		
Spiculated	38 (48.1%)		
Mass internal enhancement		5.589	0.061
Heterogeneous	65 (82.3%)		
Rim enhancement	14 (17.7%)		
NME distribution		5.391	0.068
Segmental	27 (84.3%)		
Regional or multiple regions	5 (15.6%)		
NME internal enhancement		5.024	0.081
Heterogeneous	8 (25.0%)		
Clumped or clustered ring	24 (75.0%)		
Initial phase of kinetic curve		5.262	0.154
Fast	47 (42.3%)		
Medium	51 (45.9%)		
Slow	13 (11.7%)		
Delayed phase of kinetic curve		1.041	0.791
Persistent	5 (4.5%)		
Plateau type	33 (29.7%)		
Washout	73 (65.8%)		
Assessment categories		3.439	0.329
4A	5 (4.5%)		
4B	18 (16.2%)		
4C	58 (52.3%)		
5	30 (27.0%)		

Abbreviations: EC‐wP, epirubicin/cyclophosphamide followed by weekly paclitaxel; EP‐wP, epirubicin/paclitaxel followed by weekly paclitaxel; NME, non‐mass enhancement.

^a^ Means statistically significant.

### 3.4 Predictive RS

The top three proposed models were selected: the CL‐T2‐Clinicalimaging model (Model 1), the T2_p4‐Model1 model (Model 2) and the CL_d4‐ClinicalImaging model (Model 3). Three groups of predicted RSs were generated by plugging the features of the prognosis dataset into the above three models; these RSs were denoted as model1_predRS, model2_predRS, and model3_predRS.

Model 1 predicted 79 high‐risk patients, 16 of whom had recurrence or metastasis, and the recurrence rate was 20.3% (16/79). Two of the predicted low‐risk patients developed recurrence and metastasis, for a recurrence rate of 6.3% (2/32). Model 2 and Model 3 predicted 78 high‐risk patients, 16 of whom had recurrence and metastasis, for a recurrence rate of 20.5% (16/78). Two of the predicted low‐risk patients experienced recurrence and metastasis, for a recurrence rate of 6.1% (2/33).

**FIGURE 5:**
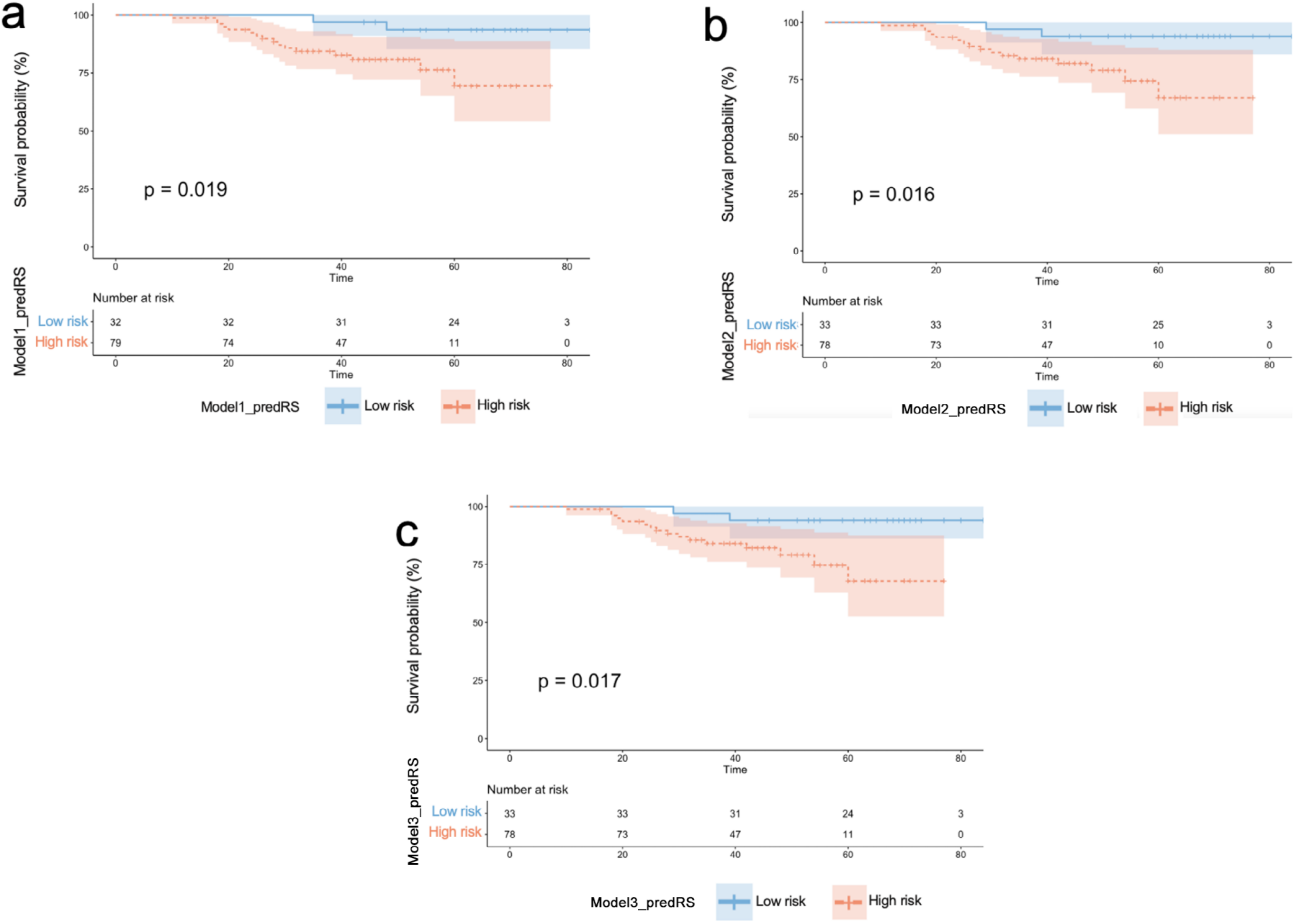
Kaplan–Meier curve for predicting recurrence risk scores (predRSs) generated by the top three models.

K–M curves of the predRSs are shown in Figure 5. The log‐rank test showed that there were significant differences between the predicted high‐risk group and the low‐risk group stratified by the three models.

### 3.5 Prognostic Assessment of Radiogenomic Signatures

**FIGURE 6:**
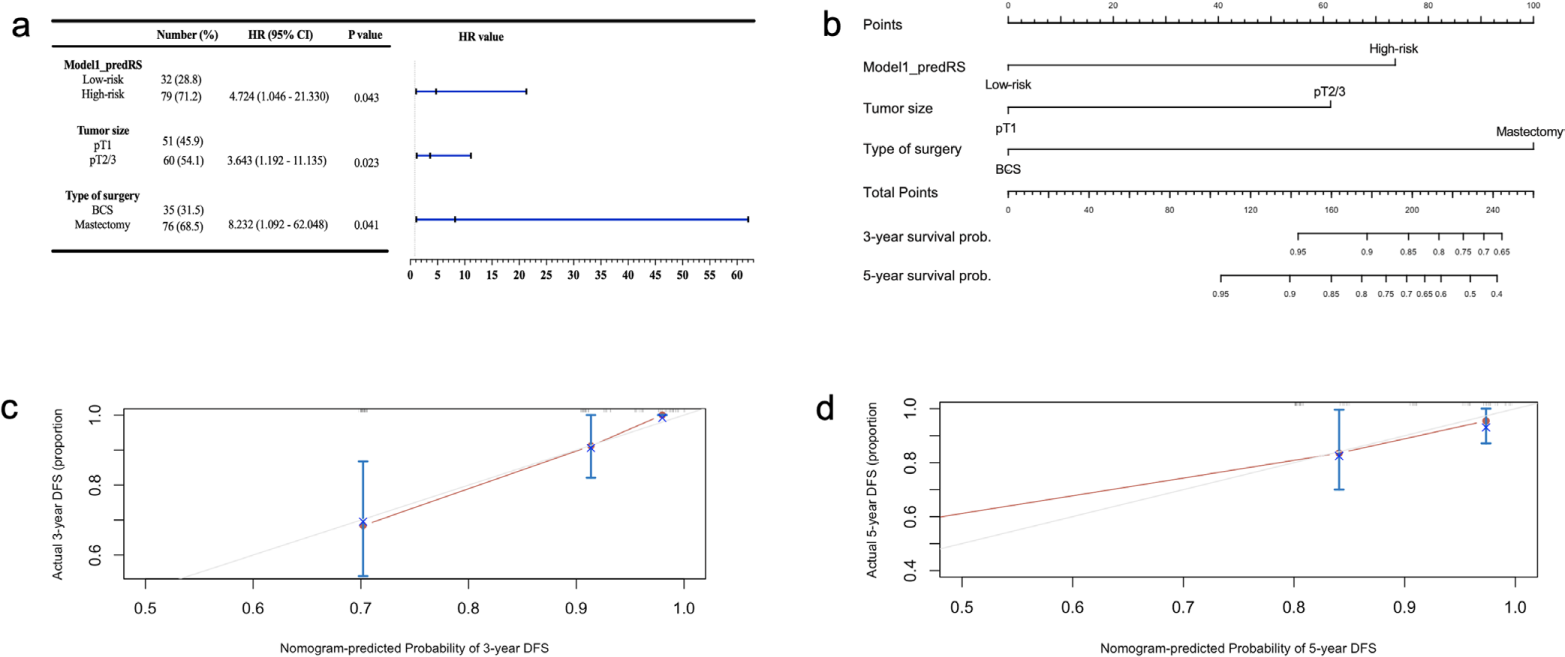
Forest plot (a), nomograph (b) and calibration curve (c, d) of the Cox proportional hazards model 1. BCS, breast conservative surgery.

Univariate log‐rank test showed that there were significant differences in lesion size, surgical type, and tumor margin in the prognosis dataset (Table 4), which along with predRS were included in Cox proportional hazards analysis (Figure 6, Figures S3 and S4).

All three Cox models showed that both predRS and surgery type had significant impacts on survival time, with recurrence or metastasis more likely to occur in patients with predicted high risk and mastectomy. The Cox1 and Cox2 models also showed that pT2/3 lesions were associated with a greater risk of recurrence or metastasis than pT1 lesions.

The nomogram showed how different factors predict 3‐ and 5‐year survival. The calibration curves indicated good agreement between the prediction and observation data for the three Cox models; the 3‐year survival prediction calibration performance was better than that of the 5‐year survival prediction.

## 4 Discussion

The National Comprehensive Cancer Network (NCCN) guidelines recommend using the 21‐gene assay to assist in the decision of whether chemotherapy is required after surgery for ER+/HER2− early breast cancer [18, 19]. However, this test is expensive and time‐consuming. In this study, a 21‐gene RS‐prediction model was constructed using radiomic features extracted from tumor and peritumoral tissues, which indicated that these radiomic features could reflect recurrence risk. Additionally, the predictive model has the potential to reflect survival after adjuvant chemotherapy in young patients with breast cancer.

We constructed tumoral prediction models using different phases of DCE and T2W images, and the CL‐T2 model performed best. Several previous studies have shown that there was a correlation between DCE radiomics characteristics and the RS [9, 14, 20, 21], I which is consistent with the findings of the present study. Moreover, compared with those of the C0‐ and CM‐based models, the CF and CL models had higher AUCs, suggesting that the vascular distribution and permeability may differ between high‐ and low‐risk lesions and that early and delayed enhancement have greater value in predicting prognosis. For T2W images, some published studies have also indicated that T2W images have potential value in evaluating the prognosis of breast cancer patients, although the molecular type and clinical stage of the patients involved are different [22, 23, 24, 25]. In a study by CHEN Y et al. [11], a T2‐based RS‐prediction model was established, similar to the results of the present study, with an AUC of 0.60. The predictive performance of the CL‐T2 model was better than that of the models based on CL or T2 alone. Several studies have also supported the use of T2W combined with DCE features, which are useful for identifying not only breast cancer but also other tumors [24, 26, 27]. After adding clinical‐imaging features to the CL‐T2 model, the performance improved, with an AUC of 0.74 in the validation group. These results indicated that there could be complementary information in CL, T2WI, and clinical‐imaging features.

In this study, multiple RS‐prediction models involving the peritumoral environment (including peritumoral and dilatation models) were established, suggesting that peritumoral features have potential value in predicting prognosis. A study published in 2021 focused on locally advanced rectal cancer showed that combining intratumoral features with peritumoral features could help to predict the efficacy of neoadjuvant therapy [28]. In terms of breast cancer, Piero et al. [10] developed a partial least square regression model by using peritumoral and intertumoral radiomic features to predict the RS, indicating the potential value of the peritumoral region. However, in addition to the small sample size (*n* = 62), this study focused only on CF and CL and never explored the value of T2W images.

This study showed that the proposed RS‐prediction model could independently affect the survival time of breast cancer patients. Fan Ming et al. [15] also established an RS‐prediction model based on MRI radiomics and found that the model was an independent predictor for evaluating the outcome after neoadjuvant therapy, which was partially consistent with the findings of the present study. To the best of our knowledge, this study is the first to investigate RS‐predictive models for evaluating survival after adjuvant chemotherapy in young patients. Moreover, this study focused not only on the tumoral radiomic features extracted from DCE images, as in Fan Ming et al.'s study [15], but also on the peritumoral and T2WI characteristics.

Our study showed that the predicted RS was positively correlated with the recurrence rate; that is, patients in the predicted high‐risk group had a higher recurrence rate than those in the low‐risk group. A study by Woodward et al. [29] showed the 10‐year local recurrence rate of low‐risk patients (RS < 18) was 9.7%, which was significantly lower than that of patients in the medium‐high‐risk group (16.5%). The research subjects in Maoli Wang et al.'s study [30] included a total of 4059 patients from the Surveillance, Epidemiology and End Results database; these patients were stratified with an RS of 11 and 25 as the cutoffs. The results showed that the RS risk groups were positively correlated with pathological and prognostic stage (*p* < 0.001), and there were significant differences in breast cancer‐specific survival and overall survival among the RS groups (*p* < 0.001). Our study is to some extent consistent with these two studies.

## 5 Limitations

The study had some limitations. First, although the validation group was set up in this study, the number of subjects in our study was limited, especially compared with numerous imaging radiomics features, and the subjects were from one center, which might cause population selection bias. Studies with larger sample sizes are needed to further explore this topic. Second, the MRI examination in this study was completed under different scanners with different scanning parameters. Although we have adopted a number of methods to improve the robustness of the model, the interscanner variability still could not be completely avoided. Third, this study did not involve DWI sequence, which was important in breast MRI, and it is necessary to carry out relevant studies on DWI prediction of RS in the future. Finally, the tumoral VOI was segmented manually and time‐consumingly. In the future, automatic or semiautomatic VOI segmentation methods, combined with automatic correction technology, probably will improve the work efficiency and repeatability of VOI segmentation.

## 6 Conclusion

In conclusion, we identified and validated 21‐gene RS‐prediction signatures based on both tumoral and peritumoral radiomics characteristics, suggesting information on the MR images could reflect the biological behavior of tumors. The proposed RS‐prediction model could provide an alternative for patients who cannot afford the 21‐gene assay or reduce the medical costs for patients who could afford it, which was an attempt to increase cost‐effectiveness as well as expand the number of patients who would benefit from the 21‐gene assay to some extent. Our study also explored extending the applicable population of the RS‐prediction model to young patients with adjuvant chemotherapy, and the results revealed that the proposed RS‐signature was associated with the 5‐year recurrence rate of them, which was an attempt to predict individual survival and assist stratification management in young patients.

## Author Contributions

**Weijun Peng:** conceptualization (equal), resources (equal). **Yang Chen:** conceptualization (equal), formal analysis (equal), writing – original draft (equal), writing – review and editing (equal). **Lizhi Xie:** conceptualization (equal), formal analysis (equal), writing – original draft (equal), writing – review and editing (equal). **Wei Tang:** conceptualization (equal), resources (equal). **Qin Xiao:** data curation (equal). **Li Liu:** data curation (equal). **Tianwen Xie:** methodology (equal). **Yan Huang:** data curation (equal). **Qifeng Wang:** methodology (equal). **Keda Yu:** conceptualization (equal), resources (equal). **Yajia Gu:** conceptualization (equal), funding acquisition (lead), resources (equal).

## Ethics Statement

The study was approved by the ethics committee of Fudan University Shanghai Cancer Center (2203252–23), and all measures of the study were in accordance with the Declaration of Helsinki guidelines.

## Consent

The informed consent requirement was waived by virtue of this study's retrospective design.

## Conflicts of Interest

The authors declare no conflicts of interest.

## Supporting information


**Table S1:** MRI Protocol.
**Table S2:** Performance of tumoral models.
**Table S3:** Performance of peritumoral models.
**Appendix S1:** Features in the peritumoral models.
**Appendix S2:** Features in the dilation models.

**Figure S1:** Features selected by different tumoral models. GLCM, gray‐level co‐occurrence matrix, GLDM, gray‐level dependence matrix; GLSZM, gray‐level size zone matrix.
**Figure S2:** Change trend in dilation model performance in the validation group. The optimal dilation models were constructed using 4 mm dilation radiomics features on the last‐enhanced (CL) phase of dynamic contrast‐enhanced (CL_d4) (a). Receiver operating characteristic (ROC) curves of the CL_d4 and the corresponding fusion models (Model 3) combined with clinical‐imaging features (b, c).
**Figure S3:** Forest plot (a), nomograph (b) and calibration curve (c, d) of the Cox proportional hazards model 2. BCS, breast conservative surgery.
**Figure S4:** Forest plot (a), nomograph (b) and calibration curve (c, d) of the Cox proportional hazards model 3. BCS, breast conservative surgery.

## Data Availability

The clinical data related to patients and MRI images can be obtained from the corresponding author on reasonable request.

## References

[1] W. J. Gradishar , M. S. Moran , J. Abraham , et al., “Breast Cancer, Version 3.2024, NCCN Clinical Practice Guidelines in Oncology,” Journal of the National Comprehensive Cancer Network 22 (2024): 331–357. 39019058 10.6004/jnccn.2024.0035

[2] R. M. Mann , C. Balleyguier , P. A. Baltzer , et al., “Breast MRI: EUSOBI Recommendations for Women's Information,” European Radiology 25 (2015): 3669–3678. 26002130 10.1007/s00330-015-3807-z PMC4636525

[3] M. A. Marino , T. Helbich , P. Baltzer , and K. Pinker‐Domenig , “Multiparametric MRI of the Breast: A Review,” Journal of Magnetic Resonance Imaging 47 (2018): 301–315. 28639300 10.1002/jmri.25790

[4] R. M. Mann , N. Cho , and L. Moy , “Breast MRI: State of the Art,” Radiology 292 (2019): 520–536. 31361209 10.1148/radiol.2019182947

[5] G. L. Menezes , F. M. Knuttel , B. L. Stehouwer , R. M. Pijnappel , and M. A. van den Bosch , “Magnetic Resonance Imaging in Breast Cancer: A Literature Review and Future Perspectives,” World Journal of Clinical Oncology 5 (2014): 61–70. 24829852 10.5306/wjco.v5.i2.61 PMC4014797

[6] N. Braman , P. Prasanna , J. Whitney , et al., “Association of Peritumoral Radiomics With Tumor Biology and Pathologic Response to Preoperative Targeted Therapy for HER2 (ERBB2)‐Positive Breast Cancer,” JAMA Network Open 2 (2019): e192561. 31002322 10.1001/jamanetworkopen.2019.2561 PMC6481453

[7] S. D. Soysal , A. Tzankov , and S. E. Muenst , “Role of the Tumor Microenvironment in Breast Cancer,” Pathobiology 82 (2015): 142–152. 26330355 10.1159/000430499

[8] G. A. Woodard , K. M. Ray , B. N. Joe , and E. R. Price , “Qualitative Radiogenomics: Association Between Oncotype DX Test Recurrence Score and BI‐RADS Mammographic and Breast MR Imaging Features,” Radiology 286 (2018): 60–70. 28885890 10.1148/radiol.2017162333

[9] H. Li , Y. T. Zhu , E. S. Burnside , et al., “MR Imaging Radiomics Signatures for Predicting the Risk of Breast Cancer Recurrence as Given by Research Versions of MammaPrint, Oncotype DX, and PAM50 Gene Assays,” Radiology 281 (2016): 382–391. 27144536 10.1148/radiol.2016152110 PMC5069147

[10] P. Chiacchiaretta , D. Mastrodicasa , A. M. Chiarelli , et al., “MRI‐Based Radiomics Approach Predicts Tumor Recurrence in ER+/HER2‐ Early Breast Cancer Patients,” Journal of Digital Imaging 36 (2023): 1071–1080. 36698037 10.1007/s10278-023-00781-5 PMC10287859

[11] Y. Chen , W. Tang , W. Liu , et al., “Multiparametric MR Imaging Radiomics Signatures for Assessing the Recurrence Risk of ER+/HER2‐ Breast Cancer Quantified With 21‐Gene Recurrence Score,” Journal of Magnetic Resonance Imaging 58 (2023): 444–453. 36440706 10.1002/jmri.28547

[12] J. Y. Kim , J. J. Kim , L. Hwangbo , et al., “Diffusion‐Weighted MRI of Estrogen Receptor‐Positive, HER2‐Negative, Node‐Negative Breast Cancer: Association Between Intratumoral Heterogeneity and Recurrence Risk,” European Radiology 30 (2020): 66–76. 31385051 10.1007/s00330-019-06383-6

[13] V. Dialani , S. Gaur , T. S. Mehta , et al., “Prediction of Low Versus High Recurrence Scores in Estrogen Receptor‐Positive, Lymph Node‐Negative Invasive Breast Cancer on the Basis of Radiologic‐Pathologic Features: Comparison With Oncotype DX Test Recurrence Scores,” Radiology 280 (2016): 370–378. 26937802 10.1148/radiol.2016151149

[14] M. A. Jacobs , C. B. Umbricht , V. S. Parekh , et al., “Integrated Multiparametric Radiomics and Informatics System for Characterizing Breast Tumor Characteristics With the OncotypeDX Gene Assay,” Cancers (Basel) 12 (2020): 2772. 32992569 10.3390/cancers12102772 PMC7601838

[15] M. Fan , Y. Cui , C. You , et al., “Radiogenomic Signatures of Oncotype DX Recurrence Score Enable Prediction of Survival in Estrogen Receptor‐Positive Breast Cancer: A Multicohort Study,” Radiology 302 (2022): 516–524. 34846204 10.1148/radiol.2021210738

[16] K. D. Yu , J. Y. Ge , X. Y. Liu , et al., “Cyclophosphamide‐Free Adjuvant Chemotherapy for Ovarian Protection in Young Women With Breast Cancer: A Randomized Phase 3 Trial,” Journal of the National Cancer Institute 113 (2021): 1352–1359. 33822134 10.1093/jnci/djab065 PMC8486325

[17] E. A. Morris , C. E. Comstock , and C. H. Lee , ACR BI‐RADS Magnetic Resonance Imaging. ACR BI‐RADS Atlas, Breast Imaging Reporting and Data System (American College of Radiology, 2013).

[18] N. L. Henry , M. R. Somerfield , V. G. Abramson , et al., “Role of Patient and Disease Factors in Adjuvant Systemic Therapy Decision Making for Early‐Stage, Operable Breast Cancer: American Society of Clinical Oncology Endorsement of Cancer Care Ontario Guideline Recommendations,” Journal of Clinical Oncology 34 (2016): 2303–2311. 27001586 10.1200/JCO.2015.65.8609

[19] L. N. Harris , N. Ismaila , L. M. McShane , et al., “Use of Biomarkers to Guide Decisions on Adjuvant Systemic Therapy for Women With Early‐Stage Invasive Breast Cancer: American Society of Clinical Oncology Clinical Practice Guideline,” Journal of Clinical Oncology 34 (2016): 1134–1150. 26858339 10.1200/JCO.2015.65.2289 PMC4933134

[20] A. B. Ashraf , D. Daye , S. Gavenonis , et al., “Identification of Intrinsic Imaging Phenotypes for Breast Cancer Tumors: Preliminary Associations With Gene Expression Profiles,” Radiology 272 (2014): 374–384. 24702725 10.1148/radiol.14131375 PMC4564060

[21] E. J. Sutton , J. H. Oh , B. Z. Dashevsky , et al., “Breast Cancer Subtype Intertumor Heterogeneity: MRI‐Based Features Predict Results of a Genomic Assay,” Journal of Magnetic Resonance Imaging 42 (2015): 1398–1406. 25850931 10.1002/jmri.24890 PMC4784421

[22] S. Kamiya , H. Satake , Y. Hayashi , et al., “Features From MRI Texture Analysis Associated With Survival Outcomes in Triple‐Negative Breast Cancer Patients,” Breast Cancer 29 (2022): 164–173. 34529241 10.1007/s12282-021-01294-1

[23] F. Chamming's , Y. Ueno , R. Ferre , et al., “Features From Computerized Texture Analysis of Breast Cancers at Pretreatment MR Imaging Are Associated With Response to Neoadjuvant Chemotherapy,” Radiology 286 (2018): 412–420. 28980886 10.1148/radiol.2017170143

[24] Z. Liu , Z. Li , J. Qu , et al., “Radiomics of Multiparametric MRI for Pretreatment Prediction of Pathologic Complete Response to Neoadjuvant Chemotherapy in Breast Cancer: A Multicenter Study,” Clinical Cancer Research 25 (2019): 3538–3547. 30842125 10.1158/1078-0432.CCR-18-3190

[25] N. L. Eun , D. Kang , E. J. Son , J. H. Youk , J. A. Kim , and H. M. Gweon , “Texture Analysis Using Machine Learning‐Based 3‐T Magnetic Resonance Imaging for Predicting Recurrence in Breast Cancer Patients Treated With Neoadjuvant Chemotherapy,” European Radiology 31 (2021): 6916–6928. 33693994 10.1007/s00330-021-07816-x

[26] H. M. Li , J. Gong , R. M. Li , et al., “Development of MRI‐Based Radiomics Model to Predict the Risk of Recurrence in Patients With Advanced High‐Grade Serous Ovarian Carcinoma,” American Journal of Roentgenology 217 (2021): 664–675. 34259544 10.2214/AJR.20.23195

[27] T. Y. Tang , X. Li , Q. Zhang , et al., “Development of a Novel Multiparametric MRI Radiomic Nomogram for Preoperative Evaluation of Early Recurrence in Resectable Pancreatic Cancer,” Journal of Magnetic Resonance Imaging 52 (2020): 231–245. 31867839 10.1002/jmri.27024 PMC7317738

[28] A. Delli Pizzi , A. M. Chiarelli , P. Chiacchiaretta , et al., “MRI‐Based Clinical‐Radiomics Model Predicts Tumor Response Before Treatment in Locally Advanced Rectal Cancer,” Scientific Reports 11 (2021): 5379. 33686147 10.1038/s41598-021-84816-3 PMC7940398

[29] W. A. Woodward , W. E. Barlow , R. Jagsi , et al., “Association Between 21‐Gene Assay Recurrence Score and Locoregional Recurrence Rates in Patients With Node‐Positive Breast Cancer,” JAMA Oncology 6 (2020): 505–511. 31917424 10.1001/jamaoncol.2019.5559 PMC6990911

[30] M. Wang , K. Wu , P. Zhang , M. Zhang , A. Ding , and H. Chen , “The Prognostic Significance of the Oncotype DX Recurrence Score in T1‐2N1M0 Estrogen Receptor‐Positive HER2‐Negative Breast Cancer Based on the Prognostic Stage in the Updated AJCC,” Annals of Surgical Oncology 26 (2019): 1227–1235. 30456680 10.1245/s10434-018-7068-3

