# Supplementary material for: MRI Radiomics Signatures of 21‐Gene Recurrence Score for Predicting Survival in ER+/HER2− Breast Cancer
Source: Cancer Med. 2025 Sep 2;14(17):e71172. doi: 10.1002/cam4.71172 (PMC12403013; doi:10.1002/cam4.71172)
Supplement: Supplementary file 1 — Table S1: MRI Protocol. Table S2: Performance of tumoral models. Table S3: Performance of peritumoral models. Appendix S1: Features in the peritumoral models. Appendix S2: Features in the dilation models. [file CAM4-14-e71172-s001.docx]

| **Table S1. MRI Protocol** | | | | | | | | | |
| --- | --- | --- | --- | --- | --- | --- | --- | --- | --- |
| **Scanner** | **Sequence** | **Scans** | **Slices** | **Slice Thickness (mm)** | **TR/TE (ms)** | **FOV**  **(mm)** | **Matrix** | **Flip Angle** | **Number of Excitations** |
| GE 1.5T (Signa HDx) | T2WI |  | 20 | 6 | 5020/45 | 330×206 | 256×160 | 90° | 1 |
|  | DCE | 5 | 52 | 2.8 | 4.8/2.3 | 240×160 | 384×256 | 15° | 0.75 |
| GE 3.0T (Signa HDx) | T2WI |  | 20 | 6 | 5420/45 | 340×212 | 256×160 | 90° | 0.5 |
|  | DCE | 5 | 100 | 3 | 4.3/2.1 | 225×150 | 384×256 | 10° | 0.7 |
| Siemens 3.0T (Skyra) | T2WI |  | 34 | 5 | 3570/69 | 350×245 | 384×269 | 80° | 1 |
|  | DCE | 6 | 80 | 2.2 | 4.5/1.56 | 360×335 | 384×357 | 10° | 1 |
| DCE = dynamic contrast enhancement; TR = time to repetition; TE = time to echo; FOV = field of view. | | | | | | | | | |

| **Table S2. ﻿﻿Performance of tumoral models** | | |  | |  |  | |  |  | |  |  |  |
| --- | --- | --- | --- | --- | --- | --- | --- | --- | --- | --- | --- | --- | --- |
| **Tumoral model** | **﻿Training Group** | | | | | | |  | **﻿Validation Group** | | | | |
|  | **AUC (95% CI)** | **﻿Accuracy** | | **﻿****Sensitivity** | | | **﻿Specificity** |  | **AUC (95% CI)** | **﻿ Accuracy** | | **﻿Sensitivity** | **﻿Specificity** |
| C0 | 0.71 (0.63-0.78) | 0.64 (0.58-0.73) | | 0.55 (0.39-0.62) | | | 0.73 (0.71-0.89) |  | 0.62 (0.44-0.77) | 0.56 (0.46-0.73) | | 0.58 (0.35-0.75) | 0.54 (0.41-0.83) |
| CF | 0.72 (0.64-0.80) | 0.64 (0.55-0.71) | | 0.64 (0.53-0.75) | | | 0.64 (0.52-0.74) |  | 0.66 (0.50-0.82) | 0.63 (0.50-0.77) | | 0.62 (0.43-0.81) | 0.62 (0.44-0.81) |
| CM | 0.70 (0.62-0.78) | 0.68 (0.62-0.76) | | 0.73 (0.56-0.76) | | | 0.64 (0.62-0.81) |  | 0.61 (0.45-0.77) | 0.58 (0.48-0.75) | | 0.62 (0.33-0.75) | 0.54 (0.48-0.86) |
| CL | 0.74 (0.65-0.81) | 0.68 (0.61-0.75) | | 0.78 (0.63-0.83) | | | 0.57 (0.53-0.74) |  | 0.65 (0.49-0.81) | 0.65 (0.44-0.71) | | 0.71 (0.38-0.77) | 0.58 (0.38-0.76) |
| DCE | 0.82 (0.75-0.88) | 0.75 (0.71-0.83) | | 0.65 (0.62-0.83) | | | 0.86 (0.73-0.90) |  | 0.61 (0.44-0.77) | 0.58 (0.46-0.73) | | 0.71 (0.63-0.95) | 0.46 (0.22-0.61) |
| T2WI | 0.68 (0.60-0.76) | 0.65 (0.58-0.73) | | 0.53 (0.45-0.67) | | | 0.77 (0.66-0.84) |  | 0.67 (0.51-0.82) | 0.63 (0.48-0.75) | | 0.67 (0.45-0.85) | 0.58 (0.35-0.75) |
| CF-T2 | 0.72 (0.64-0.80) | 0.69 (0.62-0.76) | | 0.70 (0.57-0.78) | | | 0.68 (0.60-0.80) |  | 0.64 (0.47-0.79) | 0.52 (0.46-0.73) | | 0.67 (0.44-0.82) | 0.38 (0.33-0.74) |
| CL-T2 | 0.76 (0.68-0.84) | 0.69 (0.64-0.78) | | 0.75 (0.60-0.79) | | | 0.62 (0.61-0.81) |  | 0.71 (0.53-0.85) | 0.65 (0.48-0.77) | | 0.83 (0.52-0.88) | 0.46 (0.35-0.75) |
| ClinicalImaging | 0.69 (0.61-0.76) | 0.68 (0.60-0.74) | | 0.56 (0.44-0.67) | | | 0.81 (0.69-0.88) |  | 0.68 (0.55-0.81) | 0.65 (0.50-0.77) | | 0.67 (0.48-0.85) | 0.62 (0.42-0.82) |
| CL-T2-ClinicalImaging | 0.82 (0.76-0.88) | 0.79 (0.71-0.83) | | 0.71 (0.61-0.81) | | | 0.87 (0.75-0.92) |  | 0.74 (0.59-0.87) | 0.63 (0.46-0.73) | | 0.79 (0.60-0.95) | 0.46 (0.23-0.61) |
| The pre- (C0), first- (CF), middle (CM) and last-enhanced (CL) phases of dynamic contrast-enhanced (DCE) images. | | | | | | | | | | | | | |

| **Table S3. ﻿****Performance of peritumoral models** | | | | | | | | | | |
| --- | --- | --- | --- | --- | --- | --- | --- | --- | --- | --- |
| **Peritumoral model** | **﻿Training Group** | | | |  | **﻿Validation Group** | | | | |
|  | **AUC (95% CI)** | **﻿Accuracy** | **﻿Sensitivity** | **﻿Specificity** |  | **AUC (95% CI)** | **﻿Accuracy** | **﻿Sensitivity** | | **﻿Specificity** |
| CL_p2 | 0.75 (0.66-0.82) | 0.68 (0.62-0.76) | 0.69 (0.59-0.79) | 0.68 (0.59-0.79) |  | 0.65 (0.48-0.80) | 0.56 (0.42-0.67) | 0.75 (0.52-0.88) | 0.38 (0.20-0.58) | |
| CL_p4 | 0.73 (0.65-0.81) | 0.63 (0.55-0.70) | 0.64 (0.50-0.73) | 0.62 (0.53-0.74) |  | 0.65 (0.49-0.81) | 0.44 (0.31-0.56) | 0.21 (0.05-0.39) | 0.67 (0.48-0.84) | |
| CL_p6 | 0.87 (0.81-0.92) | 0.79 (0.72-0.85) | 0.77 (0.67-0.87) | 0.81 (0.71-0.89) |  | 0.64 (0.47-0.79) | 0.63 (0.50-0.75) | 0.50 (0.30-0.70) | 0.75 (0.57-0.91) | |
| CL_p8 | 0.76 (0.69-0.84) | 0.68 (0.60-0.75) | 0.60 (0.49-0.71) | 0.77 (0.67-0.86) |  | 0.64 (0.47-0.80) | 0.56 (0.42-0.71) | 0.54 (0.35-0.73) | 0.58 (0.38-0.78) | |
| CL_p10 | 0.83 (0.76-0.89) | 0.72 (0.64-0.79) | 0.66 (0.54-0.76) | 0.78 (0.70-0.88) |  | 0.63 (0.48-0.79) | 0.58 (0.46-0.71) | 0.50 (0.30-0.69) | 0.67 (0.45-0.84) | |
| T2_p2 | 0.71 (0.62-0.79) | 0.68 (0.61-0.75) | 0.73 (0.57-0.78) | 0.64 (0.59-0.79) |  | 0.65 (0.47-0.80) | 0.65 (0.52-0.77) | 0.75 (0.50-0.88) | 0.54 (0.39-0.76) | |
| T2_p4 | 0.79 (0.72-0.86) | 0.71 (0.64-0.79) | 0.62 (0.54-0.75) | 0.81 (0.68-0.86) |  | **0.66 (0.49-0.81)** | 0.60 (0.44-0.71) | 0.67 (0.46-0.85) | 0.54 (0.30-0.71) | |
| T2_p6 | 0.76 (0.68-0.84) | 0.72 (0.65-0.79) | 0.69 (0.61-0.81) | 0.75 (0.62-0.82) |  | 0.64 (0.48-0.81) | 0.60 (0.46-0.75) | 0.71 (0.55-0.92) | 0.50 (0.25-0.65) | |
| T2_p8 | 0.73 (0.64-0.80) | 0.69 (0.63-0.77) | 0.64 (0.53-0.73) | 0.74 (0.67-0.85) |  | 0.63 (0.47-0.79) | 0.65 (0.50-0.77) | 0.83 (0.57-0.91) | 0.46 (0.30-0.72) | |
| T2_p10 | 0.71 (0.63-0.79) | 0.62 (0.57-0.72) | 0.58 (0.48-0.70) | 0.66 (0.61-0.81) |  | 0.61 (0.43-0.77) | 0.67 (0.54-0.79) | 0.79 (0.61-0.95) | 0.54 (0.33-0.74) | |
| CL_p2-T2_p2 | 0.76 (0.68-0.83) | 0.69 (0.62-0.77) | 0.74 (0.62-0.81) | 0.65 (0.56-0.77) |  | 0.63 (0.46-0.78) | 0.65 (0.52-0.77) | 0.71 (0.53-0.88) | 0.58 (0.37-0.78) | |
| CL_p4-T2_p4 | 0.84 (0.77-0.90) | 0.75 (0.68-0.82) | 0.70 (0.64-0.83) | 0.79 (0.68-0.87) |  | 0.64 (0.48-0.79) | 0.58 (0.46-0.73) | 0.83 (0.68-0.96) | 0.33 (0.16-0.52) | |
| CL_p6-T2_p6 | 0.96 (0.93-0.98) | 0.89 (0.84-0.94) | 0.83 (0.78-0.93) | 0.95 (0.86-0.97) |  | 0.61 (0.42-0.76) | 0.52 (0.38-0.65) | 0.71 (0.57-0.92) | 0.33 (0.12-0.48) | |
| CL_p8-T2_p8 | 0.81 (0.74-0.87) | 0.72 (0.64-0.78) | 0.73 (0.59-0.80) | 0.71 (0.62-0.82) |  | 0.60 (0.43-0.76) | 0.54 (0.42-0.71) | 0.62 (0.43-0.82) | 0.46 (0.29-0.70) | |
| CL_p10-T2_p10 | 0.79 (0.71-0.86) | 0.70 (0.62-0.77) | 0.69 (0.57-0.78) | 0.71 (0.61-0.82) |  | 0.59 (0.41-0.75) | 0.58 (0.44-0.73) | 0.71 (0.52-0.88) | 0.46 (0.26-0.67) | |
| CL_p2 ~ CL_p10: peritumoral models based on features extracted from 2 mm (p2), 4 mm (p4), 6 mm (p6), 8 mm (p8), 10 mm (p10) peritumoral ranges on the last-enhanced (CL) phases of dynamic contrast-enhancement (DCE) images.  T2_p2 ~ T2_p10: peritumoral models based on features extracted from 2 mm (p2), 4 mm (p4), 6 mm (p6), 8 mm (p8), 10 mm (p10) peritumoral ranges on the T2W images.  CL_p2-T2_p2 ~ CL_p10-T2_p10: peritumoral models based on features extracted from 2 mm (p2), 4 mm (p4), 6 mm (p6), 8 mm (p8), 10 mm (p10) peritumoral ranges on the CL and T2W images. | | | | | | | | | | |

**Appendix S1**

**Features in the peritumoral models**

1. **CL-p2 model (n = 6)**

CL-p2-original_firstorder_Skewness
CL-p2-original_Gray-Level Size Zone Matrix (GLSZM)_LargeAreaHighGrayLevelEmphasis
CL-p2-wavelet-HLL_GLSZM_LargeAreaHighGrayLevelEmphasis
CL-p2-wavelet-HLH_firstorder_Kurtosis
CL-p2-log-sigma-3-0-mm-3D_ Gray-Level Co-occurrence Matrix (GLCM)_Idmn
CL-p2-log-sigma-3-0-mm-3D_ Gray-Level Dependence Matrix (GLDM)_LargeDependenceHighGrayLevelEmphasis

1. **CL-p4 model (n = 2)**

CL-p4-wavelet-LHH_firstorder_Kurtosis
CL-p4-wavelet-HLH_firstorder_Kurtosis

1. **CL-p6 model (n = 9)**

CL-p6-original_shape_MajorAxisLength
CL-p6-original_shape_SurfaceVolumeRatio
CL-p6-original_GLCM_Imc1
CL-p6-wavelet-LLH_firstorder_Skewness
CL-p6-wavelet-LHL_GLCM_Idmn
CL-p6-wavelet-HLL_GLCM_Idmn
CL-p6-wavelet-HLH_firstorder_Kurtosis
CL-p6-wavelet-HLH_firstorder_Skewness
CL-p6-log-sigma-4-0-mm-3D_firstorder_Kurtosis

1. **CL-p8 model (n = 5)**

CL-p8-original_shape_SurfaceVolumeRatio
CL-p8-wavelet-LHL_GLDM_SmallDependenceLowGrayLevelEmphasis
CL-p8-wavelet-HLH_GLCM_ClusterShade
CL-p8-wavelet-HHH_GLCM_Imc1
CL-p8-log-sigma-2-0-mm-3D_GLCM_ClusterShade

1. **CL-p10 model (n = 8)**

CL-p10-original_shape_SurfaceVolumeRatio
CL-p10-wavelet-LHL_GLCM_ClusterShade
CL-p10-wavelet-LHL_GLDM_SmallDependenceLowGrayLevelEmphasis
CL-p10-wavelet-HLH_firstorder_Kurtosis
CL-p10-wavelet-HHL_firstorder_Median
CL-p10-log-sigma-2-0-mm-3D_GLCM_Imc2
CL-p10-log-sigma-2-0-mm-3D_GLCM_Idmn
CL-p10-log-sigma-2-0-mm-3D_GLDM_LargeDependenceHighGrayLevelEmphasis

1. **T2-p2 model** **(n = 3)**

T2-p2-original_shape_SurfaceVolumeRatio
T2-p2-wavelet-HHH_firstorder_Median
T2-p2-wavelet-HHH_GLDM_SmallDependenceLowGrayLevelEmphasis

1. **T2-p4 model (n = 5)**

T2-p4-wavelet-LHL_GLSZM_LargeAreaHighGrayLevelEmphasis
T2-p4-wavelet-HLL_GLCM_ClusterShade
T2-p4-wavelet-HHL_GLCM_ClusterShade
T2-p4-log-sigma-2-0-mm-3D_firstorder_Kurtosis
T2-p4-log-sigma-2-0-mm-3D_GLCM_ClusterShade

1. **T2-p6 model (n = 11)**

T2-p6-wavelet-LLL_GLCM_Correlation
T2-p6-wavelet-LLL_GLSZM_LargeAreaLowGrayLevelEmphasis
T2-p6-log-sigma-2-0-mm-3D_firstorder_Skewness
T2-p6-log-sigma-3-0-mm-3D_GLSZM_GrayLevelNonUniformity
T2-p6-log-sigma-3-0-mm-3D_GLSZM_LargeAreaHighGrayLevelEmphasis
T2-p6-log-sigma-3-0-mm-3D_GLDM_DependenceVariance
T2-p6-log-sigma-4-0-mm-3D_firstorder_Mean
T2-p6-log-sigma-4-0-mm-3D_GLCM_Correlation
T2-p6-log-sigma-4-0-mm-3D_GLCM_Imc1
T2-p6-log-sigma-4-0-mm-3D_GLCM_Idmn
T2-p6-log-sigma-4-0-mm-3D_GLDM_LargeDependenceLowGrayLevelEmphasis

1. **T2-p8 model (n = 6)**

T2-p8-original_shape_Elongation
T2-p8-original_shape_Flatness
T2-p8-original_shape_MajorAxisLength
T2-p8-original_shape_Sphericity
T2-p8-original_shape_SurfaceVolumeRatio
T2-p8-log-sigma-4-0-mm-3D_GLDM_DependenceVariance

1. **T2-p10 model (n = 3)**

T2-p10-original_shape_Sphericity
T2-p10-wavelet-HLH_firstorder_Skewness
T2-p10-wavelet-HLH_GLCM_Idmn

1. **CL-p2 + T2-p2 model (n = 5)**

CL-p2-wavelet-LHL_GLCM_Idmn
CL-p2-wavelet-HLH_firstorder_Kurtosis
CL-p2-wavelet-HHL_GLCM_Correlation
CL-p2-wavelet-HHL_GLCM_Imc1
T2-p2-wavelet-HHH_firstorder_Median

1. **CL-p4 + T2-p4 model (n = 4)**

CL-p4-wavelet-LHL_GLDM_LargeDependenceHighGrayLevelEmphasis
CL-p4-wavelet-HLH_GLCM_Idmn
T2-p4-wavelet-HHH_firstorder_Median
T2-p4-wavelet-HHH_firstorder_Skewness

1. **CL-p6 + T2-p6 model (n = 6)**

CL-p6-wavelet-LHH_GLDM_LargeDependenceHighGrayLevelEmphasis
CL-p6-wavelet-HLH_GLDM_LargeDependenceHighGrayLevelEmphasis
T2-p6-original_shape_Sphericity
T2-p6-wavelet-HLH_firstorder_Skewness
T2-p6-wavelet-HHH_GLCM_Idn
T2-p6-log-sigma-3-0-mm-3D_GLDM_DependenceVariance

1. **CL-p8 + T2-p8 model (n = 10)**

CL-p8-wavelet-LLH_firstorder_Kurtosis
CL-p8-wavelet-LHL_GLCM_ClusterShade
CL-p8-wavelet-LHL_GLCM_Idmn
CL-p8-log-sigma-2-0-mm-3D_GLDM_LargeDependenceHighGrayLevelEmphasis
CL-p8-log-sigma-3-0-mm-3D_firstorder_Skewness
CL-p8-log-sigma-4-0-mm-3D_firstorder_Kurtosis
T2-p8-wavelet-LHL_GLSZM_ZoneEntropy
T2-p8-wavelet-HLH_GLCM_Idmn
T2-p8-wavelet-LLL_GLSZM_LargeAreaLowGrayLevelEmphasis
T2-p8-log-sigma-2-0-mm-3D_firstorder_Kurtosis

1. **CL-p10 + T2-p10 model (n = 5)**

CL-p10-original_GLCM_Imc2
CL-p10-wavelet-LHL_GLCM_ClusterShade
CL-p10-log-sigma-3-0-mm-3D_firstorder_Skewness
CL-p10-log-sigma-4-0-mm-3D_firstorder_Kurtosis
T2-p10-log-sigma-4-0-mm-3D_GLSZM_ZoneVariance

**Appendix S2**

**Features in the dilation models**

1. **CL-d2 model (n = 11)**

CL-d2-original_Gray-Level Co-occurrence Matrix (GLCM)_ClusterShade
CL-d2-wavelet-LLH_firstorder_Mean
CL-d2-wavelet-LLH_firstorder_Median
CL-d2-wavelet-LHL_firstorder_Median
CL-d2-wavelet-LHH_firstorder_Kurtosis
CL-d2-wavelet-LHH_firstorder_Median
CL-d2-wavelet-LHH_GLCM_Idmn
CL-d2-wavelet-HLH_GLCM_Imc1
CL-d2-wavelet-HHL_GLCM_Correlation
CL-d2-wavelet-HHH_Gray-Level Dependence Matrix (GLDM)_SmallDependenceLowGrayLevelEmphasis
CL-d2-log-sigma-2-0-mm-3D_GLCM_Idn

1. **CL-d4 model (n = 10)**

CL-d4-wavelet-HHH_GLDM_SmallDependenceLowGrayLevelEmphasis
CL-d4-wavelet-LLL_firstorder_Kurtosis
CL-d4-wavelet-LLL_firstorder_Minimum
CL-d4-wavelet-LLL_GLCM_Idn
CL-d4-log-sigma-2-0-mm-3D_firstorder_Median
CL-d4-log-sigma-3-0-mm-3D_GLCM_Correlation
CL-d4-log-sigma-3-0-mm-3D_GLCM_Idn
CL-d4-log-sigma-3-0-mm-3D_GLDM_LargeDependenceHighGrayLevelEmphasis
CL-d4-log-sigma-3-0-mm-3D_GLDM_LargeDependenceLowGrayLevelEmphasis
CL-d4-log-sigma-4-0-mm-3D_firstorder_Kurtosis

1. **CL-d6 model (n = 6)**

CL-d6-original_shape_MajorAxisLength
CL-d6-wavelet-LHL_GLCM_Idmn
CL-d6-wavelet-LHH_GLDM_LargeDependenceHighGrayLevelEmphasis
CL-d6-wavelet-HHL_firstorder_Median
CL-d6-wavelet-HHL_firstorder_Median
CL-d6-log-sigma-4-0-mm-3D_firstorder_Kurtosis

1. **CL-d8 model (n = 11)**

CL-d8-original_firstorder_10Percentile
CL-d8-wavelet-LLH_firstorder_Kurtosis
CL-d8-wavelet-LLH_firstorder_Mean
CL-d8-wavelet-LHL_GLCM_Idmn
CL-d8-wavelet-LHH_firstorder_Median
CL-d8-wavelet-HLL_firstorder_Kurtosis
CL-d8-wavelet-HHH_GLCM_ClusterShade
CL-d8-wavelet-HHH_GLCM_Imc1
CL-d8-wavelet-HHH_GLDM_DependenceNonUniformityNormalized
CL-d8-log-sigma-3-0-mm-3D_firstorder_Median
CL-d8-log-sigma-4-0-mm-3D_GLDM_SmallDependenceLowGrayLevelEmphasis

1. **CL-d10 model (n = 5)**

CL-d10-wavelet-LHL_GLCM_ClusterShade
CL-d10-wavelet-LHL_GLCM_Idmn
CL-d10-wavelet-LHH_GLDM_LargeDependenceHighGrayLevelEmphasis
CL-d10-wavelet-HHH_firstorder_Median
CL-d10-log-sigma-4-0-mm-3D_GLDM_SmallDependenceLowGrayLevelEmphasis

1. **T2-d2 model (n = 10)**

T2-d2-original_shape_SurfaceVolumeRatio
T2-d2-wavelet-LLH_GLCM_Idmn
T2-d2-wavelet-LHH_firstorder_Mean
T2-d2-wavelet-HLL_firstorder_Median
T2-d2-wavelet-HLL_GLCM_Idmn
T2-d2-wavelet-HLH_firstorder_Skewness
T2-d2-wavelet-HHL_firstorder_Mean

T2-d2-wavelet-HHH_firstorder_Median
T2-d2-wavelet-HHH_GLCM_Idn
T2-d2-wavelet-HHH_GLDM_SmallDependenceLowGrayLevelEmphasis

1. **T2-d4 model (n = 9)**

T2-d4-original_shape_Elongation
T2-d4-wavelet-LLH_firstorder_Mean
T2-d4-wavelet-LLH_Gray-Level Size Zone Matrix (GLSZM)_ZoneVariance
T2-d4-wavelet-HLL_firstorder_Median
T2-d4-wavelet-HLH_firstorder_Skewness
T2-d4-wavelet-HHH_firstorder_Median
T2-d4-wavelet-HHH_GLCM_Idn
T2-d4-log-sigma-3-0-mm-3D_firstorder_Median
T2-d4-log-sigma-4-0-mm-3D_GLCM_Idmn

1. **T2-d6 model (n = 4)**

T2-d6-wavelet-HLL_firstorder_Kurtosis
T2-d6-wavelet-HLL_firstorder_Skewness
T2-d6-wavelet-HLH_firstorder_Skewness
T2-d6-wavelet-HHH_GLCM_Idn

1. **T2-d8 model (n = 3)**

T2-d8-original_GLCM_Imc1
T2-d8-wavelet-HLH_firstorder_Skewness
T2-d8-wavelet-LLL_GLCM_Correlation

1. **T2-d10 model (n = 4)**

T2-d10-wavelet-LHL_GLCM_Correlation
T2-d10-wavelet-HLH_firstorder_Skewness
T2-d10-wavelet-HHL_GLCM_Idmn
T2-d10-log-sigma-4-0-mm-3D_GLCM_Imc1

1. **CL-d2 + T2-d2 model (n = 10)**

CL-d2-wavelet-LHH_GLCM_Idmn
CL-d2-wavelet-HLH_firstorder_Kurtosis
CL-d2-wavelet-HHH_GLCM_Idn
CL-d2-wavelet-LLL_firstorder_Minimum
CL-d2-log-sigma-4-0-mm-3D_firstorder_90Percentile
T2-d2-wavelet-LLH_GLCM_Idmn
T2-d2-wavelet-HLH_firstorder_Skewness
T2-d2-wavelet-HHL_GLDM_SmallDependenceLowGrayLevelEmphasis
T2-d2-wavelet-HHH_GLCM_Idn
T2-d2-wavelet-HHH_GLDM_SmallDependenceLowGrayLevelEmphasis

1. **CL-d4 + T2-d4 model (n = 11)**

CL-d4-wavelet-LLL_firstorder_Kurtosis
CL-d4-wavelet-LLL_GLCM_Idn
CL-d4-log-sigma-3-0-mm-3D_GLCM_Idn
CL-d4-log-sigma-4-0-mm-3D_firstorder_Kurtosis
T2-d4-original_shape_Elongation
T2-d4-wavelet-LLH_firstorder_Mean
T2-d4-wavelet-LLH_GLSZM_ZoneVariance
T2-d4-wavelet-HLH_firstorder_Skewness
T2-d4-wavelet-HHH_firstorder_Median
T2-d4-wavelet-HHH_GLCM_Idn
T2-d4-log-sigma-4-0-mm-3D_GLCM_Idmn

1. **CL-d6 + T2-d6 model (n = 10)**

CL-d6-wavelet-LHL_GLCM_Idmn
CL-d6-wavelet-LHH_firstorder_Mean
CL-d6-wavelet-LHH_firstorder_Skewness
CL-d6-wavelet-HLL_GLDM_SmallDependenceLowGrayLevelEmphasis
CL-d6-wavelet-HLH_firstorder_Mean
CL-d6-wavelet-HHL_firstorder_Median
CL-d6-wavelet-HHH_firstorder_Mean
CL-d6-wavelet-LLL_GLCM_Idn
CL-d6-log-sigma-2-0-mm-3D_GLCM_Idmn
T2-d6-wavelet-LLH_firstorder_Median

1. **CL-d8 + T2-d8 model (n = 11)**

CL-d8-wavelet-LHL_GLCM_ClusterShade
CL-d8-wavelet-LHL_GLCM_Idmn
CL-d8-wavelet-LHH_GLDM_LargeDependenceHighGrayLevelEmphasis
CL-d8-wavelet-HLL_GLCM_Imc2
CL-d8-wavelet-HLL_GLDM_SmallDependenceLowGrayLevelEmphasis
CL-d8-wavelet-HHH_GLCM_ClusterShade
CL-d8-wavelet-HHH_GLCM_Imc1
CL-d8-wavelet-HHH_GLDM_DependenceNonUniformityNormalized
CL-d8-log-sigma-4-0-mm-3D_GLCM_Correlation
T2-d8-wavelet-LHL_GLCM_Correlation
T2-d8-wavelet-HHL_GLDM_SmallDependenceLowGrayLevelEmphasis

1. **CL-d10 + T2-d10 model (n = 5)**

CL-d10-log-sigma-4-0-mm-3D_GLDM_SmallDependenceLowGrayLevelEmphasis
T2-d10-wavelet-LHL_GLCM_Correlation
T2-d10-wavelet-HLH_firstorder_Skewness
T2-d10-wavelet-HHL_GLCM_Idmn
T2-d10-log-sigma-4-0-mm-3D_GLCM_Imc1
