# Supplementary material for: MRI Radiomics Signatures of 21‐Gene Recurrence Score for Predicting Survival in ER+/HER2− Breast Cancer
Source: Cancer Med. 2025 Sep 2;14(17):e71172. doi: 10.1002/cam4.71172 (PMC12403013; doi:10.1002/cam4.71172)
Supplement: Supplementary file 2 — Figure S1: Features selected by different tumoral models. GLCM, gray‐level co‐occurrence matrix, GLDM, gray‐level dependence matrix; GLSZM, gray‐level size zone matrix. Figure S2: Change trend in dilation model performance in the validation group. The optimal dilation models were constructed using 4 mm dilation radiomics features on the last‐enhanced (CL) phase of dynamic contrast‐enhanced (CL_d4) (a). Receiver operating characteristic (ROC) curves of the CL_d4 and the corresponding fusion models (Model 3) combined with clinical‐imaging features (b, c). Figure S3: Forest plot (a), nomograph (b) and calibration curve (c, d) of the Cox proportional hazards model 2. BCS, breast conservative surgery. Figure S4: Forest plot (a), nomograph (b) and calibration curve (c, d) of the Cox proportional hazards model 3. BCS, breast conservative surgery. [file CAM4-14-e71172-s002.docx]

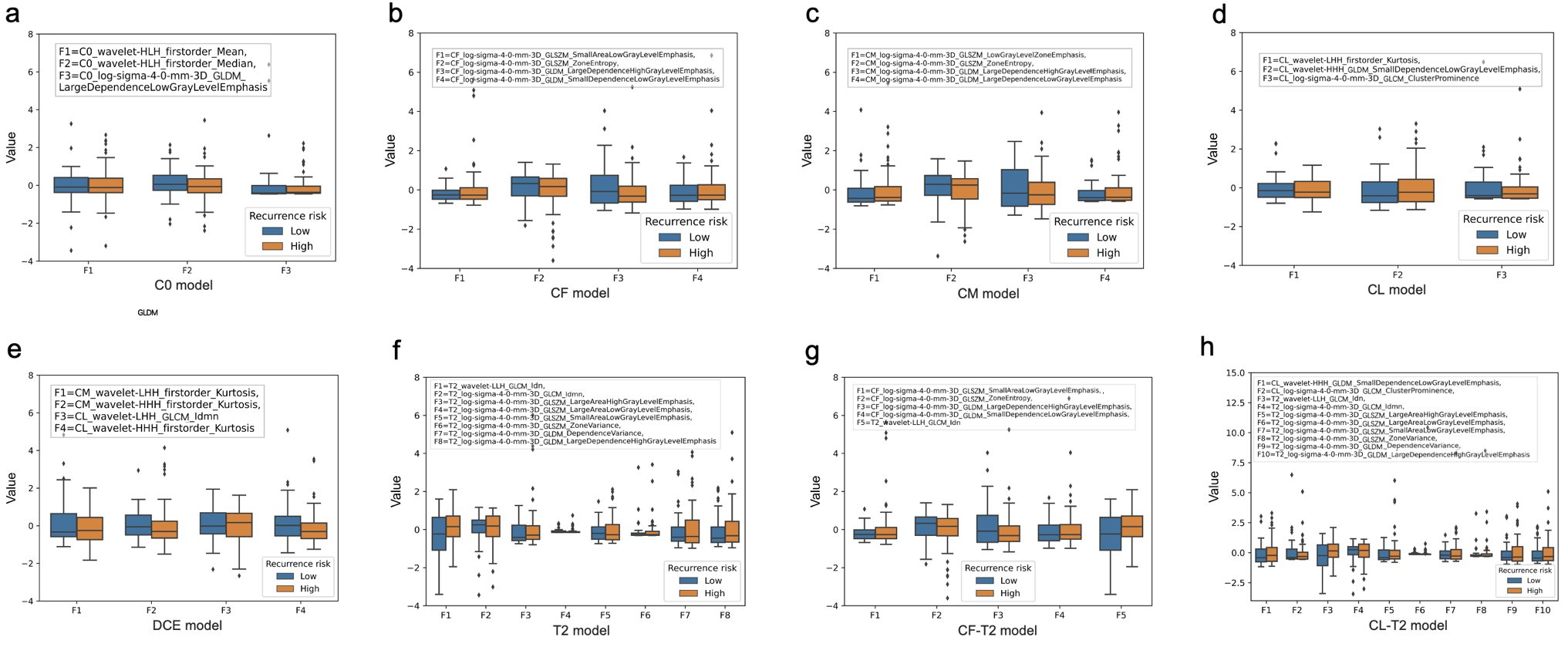


**FIGURE S1.** Features selected by different tumoral models. GLCM = Gray-Level Co-occurrence Matrix, GLDM = Gray-Level Dependence Matrix and GLSZM = Gray-Level Size Zone Matrix.


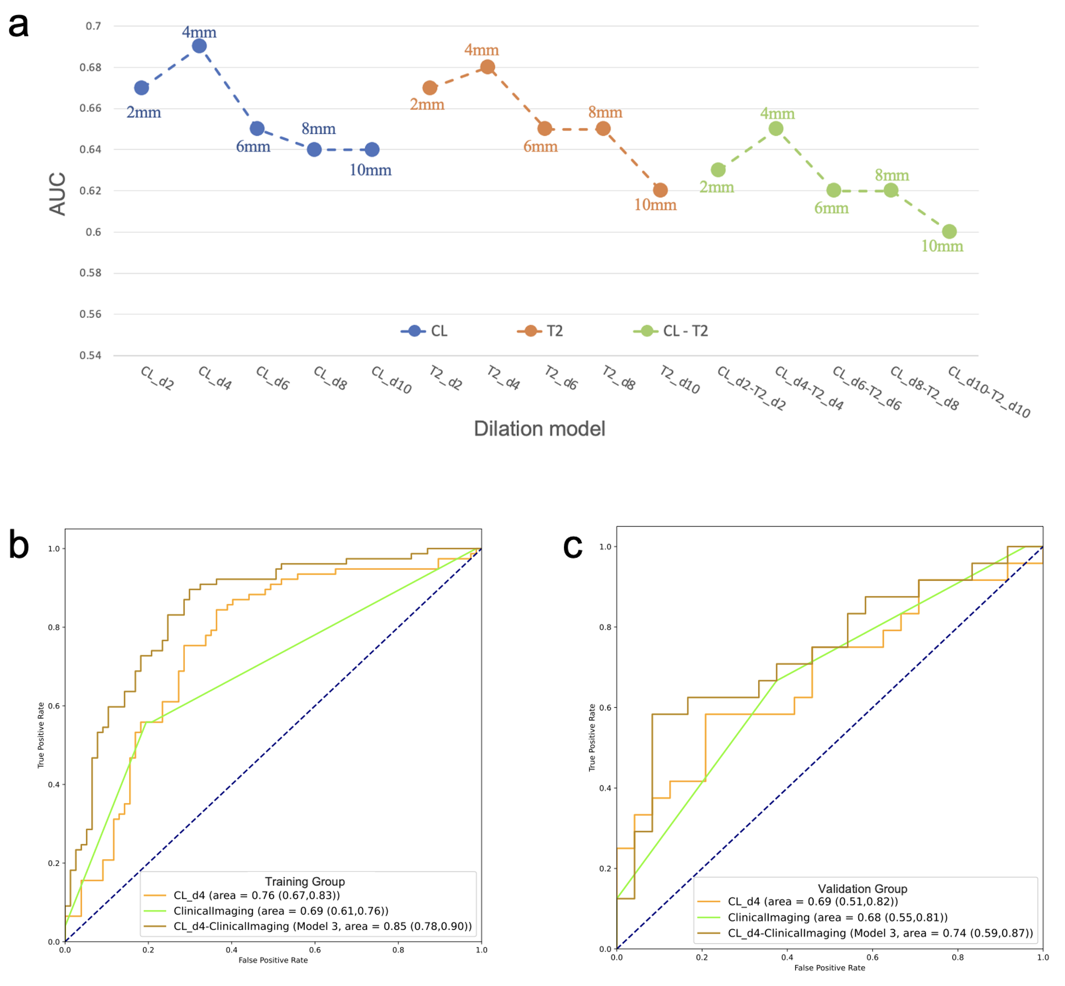


**FIGURE S2.** Change trend in dilation model performance in the validation group. The optimal dilation models were constructed using 4 mm dilation radiomics features on the last-enhanced (CL) phase of dynamic contrast-enhanced (CL_d4) (a). Receiver operating characteristic (ROC) curves of the CL_d4 and the corresponding fusion models (Model 3) combined with clinical-imaging features (b and c).


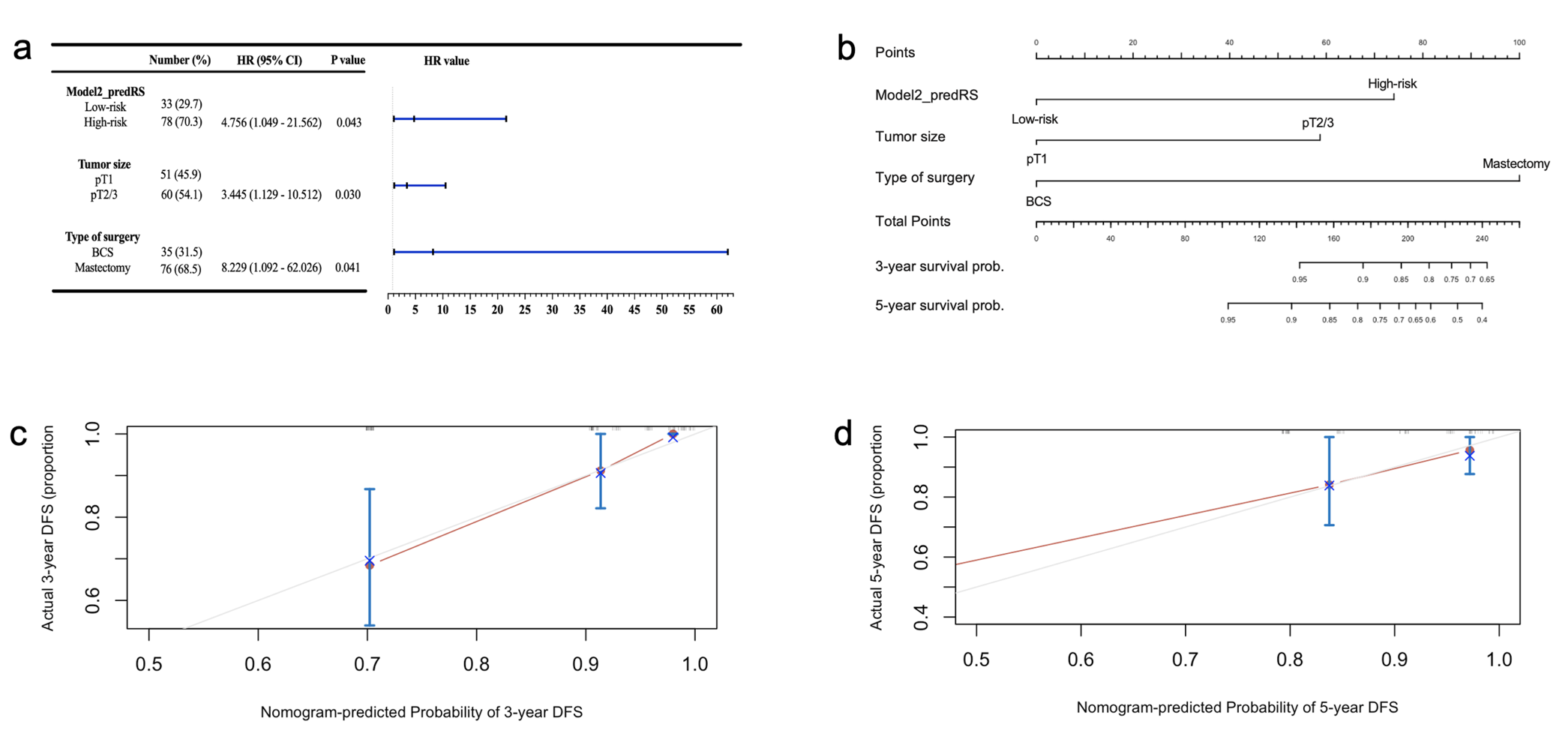


**FIGURE S3.** Forest plot (a), nomograph (b) and calibration curve (c and d) of the Cox proportional hazards model 2. BCS = breast conservative surgery.


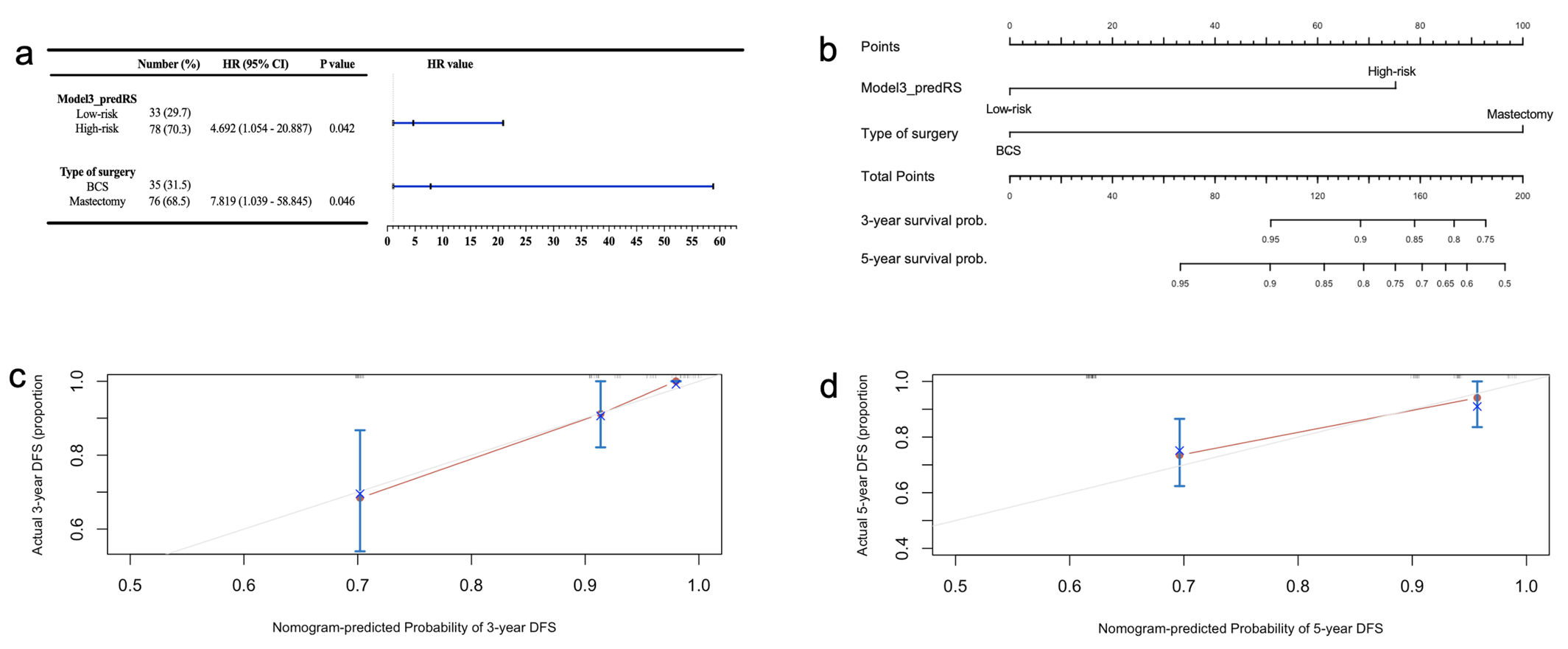


**FIGURE S4.** Forest plot (a), nomograph (b) and calibration curve (c and d) of the Cox proportional hazards model 3. BCS = breast conservative surgery.
